# Myeloid cell-specific β2-adrenergic receptor deletion improves early cardiac injury resolution via de-repression of Anxa1

**DOI:** 10.7150/thno.123651

**Published:** 2026-01-14

**Authors:** Tapas K. Nayak, Anamika Bajpai, Viren Patwa, Rhonda L. Carter, Nitya Enjamuri, Erhe Gao, Sudarsan Rajan, Yang K. Xiang, Douglas G. Tilley

**Affiliations:** 1 Aging + Cardiovascular Discovery Center, Lewis Katz School of Medicine, Temple University, Philadelphia, PA, USA, 19140.; 2 Department of Medicine, University of California at Los Angeles, CA, USA, 90095.

## Abstract

**Rationale:** Myeloid cells, including neutrophils (Nu), monocytes (Mo) and macrophages (Mac), rapidly accumulate after ischemic cardiac injury where they play integral roles in inflammation and repair. β2-adrenergic receptor (β2AR) signaling regulates numerous facets of immune cell behavior, but its impact on myeloid cell-specific responses to acute cardiac injury is unclear.

**Methods:** Myeloid cell-specific β2AR knockout (LB2) and control mice were subjected to myocardial infarction (MI) with or without prior transplantation with shRNA-modified bone marrow. The impact of myeloid cell-specific β2AR deletion, and mechanistic basis for the effect, were assessed via echocardiography, immunohistochemistry, flow cytometry, gene expression and efferocytosis analyses.

**Results:** LB2 mice displayed better cardiac function and less fibrotic remodeling post-MI. Despite a similar initial influx of myeloid cell subtypes, by 4 days post-MI LB2 mice had significantly reduced Nu, concurrent with increased Nu-containing Mac, indicating an enhanced efferocytosis capacity. Indeed, the pro-efferocytotic protein annexin A1 (AnxA1) was elevated in several β2AR-deficient myeloid cell types following MI. Mechanistically, we found the expression of several miRs known to repress *Anxa1* expression were elevated in response to β2AR stimulation, an effect absent with β2AR deletion, and miR-374b-5p mimic in particular was sufficient to decrease *Anxa1* expression. Finally, lentivirus-encoded shRNA was used to induce knockdown of *Anxa1* expression in the bone marrow of LB2 mice prior to MI, which reduced Nu efferocytosis *in vitro* and prevented the ameliorative effects on cardiac fibrosis and function observed with LB2 mice following MI *in vivo*.

**Conclusion:** Myeloid cell-specific β2AR deletion leads to loss of miR-374b-5p-mediated repression of AnxA1, which allows for enhanced efferocytosis-mediated Nu clearance, thereby limiting infarct expansion and improving post-injury cardiac function and fibrotic remodeling.

## Introduction

Acute ischemic injury, such as myocardial infarction (MI), is a major contributor of cardiovascular disease, a leading cause of mortality in the U.S., which affects millions of patients and costs billions of dollars annually 1, 2. Myeloid cells, including neutrophils (Nu), monocytes (Mo) and macrophages (Mac), normally constitute a small fraction of the adult heart but play major roles in the response to acute ischemic injury, scavenging dead cells and secreting numerous factors such as cytokines and chemokines to promote the accumulation of additional inflammatory cells 3-9. Myeloid cells perform several functions including efferocytosis (receptor-mediated phagocytosis), the clearing of dead cells and debris from a site of injury 10, 11. Nu clearance by Mac from the infarct site in the heart is critical for inflammation resolution and scar formation since the presence of dead/dying aged Nu triggers secondary inflammation that can lead to further damage and tissue necrosis 12, 13. Mac-dependent efferocytosis is one of the dominant mechanisms of Nu clearance and can be mediated by several receptor-ligand interactions. Recent work has detailed the importance of efferocytosis following cardiac injury and some of the key molecular players involved, including annexin A1 (Anxa1) 9, 14-16 . Indeed, several studies have demonstrated that myeloid cell (Nu or Mac)-produced Anxa1 acts to enhance Nu apoptosis and subsequent Mac-mediated efferocytosis to resolve inflammation 4, 17-22. Ultimately, more efficient clearance of apoptotic Nu/debris leads to better cardiac function and remodeling outcomes.

As reports accumulate each year better defining myeloid cell subpopulations and their plasticity within the heart, either normally or following injury, their potential for modulation toward promoting improved cardiac function and remodeling outcomes continues to grow. Myeloid cells express an extensive repertoire of cell surface receptors that regulate their function and responsiveness to injury, including G protein-coupled receptors (GPCRs), stimulation of which can induce rapid alterations in cell polarity, chemotaxis, adhesion and migration 23-25. Chemokine GPCRs in particular have been a major focus as regulators of immune cells, however many other receptor types, including neurohormone GPCRs, are expressed on myeloid cells and can impact their function 26. Indeed, the β2-adrenergic receptor (β2AR) is expressed on numerous immune cell types and has been shown to regulate an array of functions, including inflammation, egress, migration and immunosuppression 27-31.

We previously reported that β2AR regulates generalized immune cell recruitment to the heart following acute injury 32, 33, however the impact of β2AR specifically in myeloid cells, as the first responders to acute cardiac injury, is unclear. To directly investigate this, we crossed floxed β2AR and constitutive myeloid cell-expressing Cre (LysM-Cre) mice to generate myeloid cell-specific β2AR knockout mice. Here we demonstrate that these mice experience enhanced injury resolution through enhanced Nu efferocytosis early after injury, resulting in decreased maladaptive cardiac remodeling over time. Additionally, we show that the increased efferocytosis observed in β2AR-deficient myeloid cells is Anxa1-dependent, which β2AR normally represses under conditions of enhanced catecholamines via upregulation of miR-374b-5p. Accordingly, decreasing Anxa1 expression in β2AR knockout bone marrow cells normalized both *in vitro* efferocytosis and *in vivo* cardiac remodeling responses. Thus, myeloid cell-specific β2AR deletion improves post-ischemic injury-induced cardiac function and fibrotic remodeling via enhanced AnxA1-dependent efferocytosis-mediated Nu clearance.

## Materials and Methods

### Animal studies

Experiments using mice were conducted under the National Institutes of Health (NIH) Guide for the Care and Use of Laboratory Animals and as approved by the Institutional Animal Care and Use Committee (IACUC) at Temple University with Animal Protocols 4891 and 4902. β2AR floxed mice (FL/FL) 34 were a gift from Dr Gerard Karsenty (Columbia University) and were backcrossed onto the C57BL/6J background for 7 generations prior to crossbreeding with LysMcre (LMC) mice (#004781; The Jackson Laboratory, ME, USA) to develop myeloid cell-specific β2AR knockout mice (LB2). All mice were housed with ad libitum food and water and genotyped using flox site- and Cre-specific primers before experimental procedures (Table S1).

### Coronary artery occlusion surgery

Both male and female mice (12-14 weeks old) were used for experimental myocardial infarction (MI) as reported previously 32, 33, 35. Briefly, mice were anesthetized with 3% isoflurane inhalation, after which a small skin incision was made over the left chest, and a purse suture was made, allowing the pectoral muscles to be dissected and retracted. The fourth intercostal space was then exposed, and a small hole was made to gently extract the heart. Once the left main descending coronary artery (LCA) was located, it was sutured and ligated approximately 3 mm from its origin with a 6-0 silk suture, after which the heart was placed back into the intra-thoracic space and the chest was closed. Sham-operated mice were treated similarly, except the LCA was not occluded. Immediately after surgery, all mice received a single subcutaneous dose of buprenorphine at 0.3 mg/kg for pain alleviation.

### Echocardiography

Cardiac function was assessed by transthoracic 2-dimentional M-mode (short axis) echocardiography using the VisualSonic Vevo 2100 or 3100 Imaging System (FUJIFILM, Toronto, ON) as reported previously 32, 36. Prior to echocardiography, chest fur was removed by Nair for clear image acquisition. Mice were anesthetized by 3% isoflurane inhalation and maintained between 1.0-1.5% throughout the imaging procedure. Echocardiography LV-trace data analysis was performed by using Vevo Lab 5.5.0 software.

### Lentivirus transduction and bone marrow transplant (BMT)

Bone marrow cells from the femurs of LB2 mice were isolated and transduced with either scrambled or Annexin A1 (Anxa1) shRNA using TR30030 pRFP-C-shLenti vector (Origene Technologies Inc. MD, USA) at an MOI of 150 (Table S2). Lentivirus transduction was performed in serum free RPMI-1640 (w/o antibiotic and antimycotic) in the presence of ViralEntry™ Transduction Enhancer (Applied Biological Materials Inc. ABM, BC, Canada). A non-effective shRNA cassette in the TR30030 pRFP-C-shLenti vector plasmid was used as a scrambled control. After 2 h, cells were washed in 1x PBS and injected immediately to irradiated mice. 8-week-old LB2 male mice were irradiated using RS-2000 X-ray irradiator with 950 rads to deplete recipient bone marrow cells. Sex- and age-matched donor LB2 mouse bone marrow cells infected with lentivirus (as described above) were adoptively transferred (10 x 10^6^ live cells/mouse) into isoflurane-anesthetized mice within 24 h of irradiation via retroorbital injection. Immediately after the procedure, all BMT recipient mice received a single sub-Q dose of buprenorphine at 0.3 mg/kg to alleviate pain. All the animals were allowed to reconstitute the bone marrow for 1-month prior experiments. Upon the termination of the experiments, the success of BM cell reconstitution was confirmed for each mouse by RT-qPCR.

### Tissue processing for Masson's trichrome staining

Excised hearts were collected and perfused with 1X PBS containing 5 mM of EDTA and 60 mM potassium chloride (KCl) on ice for diastolic heart arrest. Hearts were fixed in an excess volume of 4% paraformaldehyde, paraffin embedded and sectioned at a thickness of 5 µM. Deparaffinized and rehydrated sections were processed for Masson's trichrome staining (Sigma-Aldrich, USA). 20X images were attained on a Nikon Eclipse microscope. Infarct sizes were calculated using NIS elements and normalized to LV length. To assess border zone fibrosis, 20X images were attained starting from the solid blue (infarct) area up until the solid red (remote zone) area. Each image was quantified in ImageJ as % blue staining per image area, with all images averaged to provide the % border zone fibrosis per heart. Infarct sizes were calculated using NIS elements software and interstitial fibrosis was calculated using ImageJ software.

### TTC (2,3,5-Triphenyltetrazolium) staining

Excised hearts were perfused as described under Masson's Trichrome staining and were frozen for 2 h in a 35 mm petri dish. Using an industrial blade, semi-frozen hearts were sliced into five equal sections perpendicular to the long axis of the heart and incubated in freshly prepared 1% TTC solution at 37 °C. After 30 min, sections were rinsed in 1X PBS at room temperature and the fourth section from the apex of the heart was imaged using a Nikon SMZ1000 microscope at 0.8X magnification with a dark paper underneath. Percent infarct area was quantified using ImageJ software.

### Heart perfusion for digestion

Prior to excision, hearts were perfused with excess of cold 1X PBS (containing 5 mM of EDTA and 60 mM KCl) to reduce vascular immune cell contamination 37. The heart was harvested and stored in RMPI-1460 on ice until tissue processing. Using an industrial blade, the heart was minced ~1 mm^3^ in size (and until a smooth consistency was observed) and collected in a 50 mL falcon tube containing digestion buffer composed of Collagenase type-2 (450 U/mL), Hyaluronidase (60 U/mL) and DNase I (60 U/mL) in serum-free RPMI-1640. The digestion was carried out in an incubator set at 37 °C with gentle horizontal shanking for 1 h. FBS was added to a final concentration of 10% (v/v) and the digested tissues were passed through 40 µM cell strainer. The homogenized samples were centrifuged at 400 x g for 5 min at 4 °C and cell pellets were subjected to 1X ACK lysing buffer for 2 min to remove RBC contamination. Once RBC lysis was complete, 2 volumes of 1X PBS were added and centrifuged as above. The resultant cells were resuspended in complete RPMI-1640 and stored at 4 °C until further use.

### Cell culture and primary cell isolation

L929 and Jurkat cells were maintained in complete Dulbecco′s Modified Eagle′s Medium (DMEM) or RPMI-1640 supplemented with penicillin, streptomycin, and amphotericin-B (PSF) and 10% Fetal bovine serum (FBS) at 37 °C under a humidified incubator with 5% CO_2_ respectively. Cell culture supernatants containing M-CSF were collected twice a week, passed through 0.22 µM sterile filter and stored at -20 °C until use for macrophage differentiation.

Mouse bone marrow cells were isolated as described by others 38, 39. Briefly, both femurs and tibias were flushed with RPMI-1640 supplemented with 2 mM EDTA and cells were collected in a 50 mL falcon tube. Cells were centrifuged at 300 x g for 5 min at room temperature (RT). Red blood cells (RBC) were lysed using 1X Ammonium-Chloride-Potassium (ACK) lysing buffer followed by filtered through 40 µM cell strainer and were spun down as above. The resultant cell pellets were resuspended in respective media as per downstream applications. For bone marrow-derived macrophage (BMDM) culture, cells were resuspended in complete RPMI-1640 containing 20% L929 conditioned media at a seeding density of 10 x 10^6^ cells/100 mm dish. After 4 days, media was replaced, and cells were cultured for another 2 days. On day 6, BMDM were detached by 2 mM EDTA treatment and re-seeded in tissue culture plates as per downstream experiments.

### Radioligand binding assay

The density of beta-adrenergic receptor (βAR) on the membranes of bone marrow derived macrophages (BMDM) was determined by saturation binding experiment as previously described 40. Frozen 150 mm dishes of BMDMs were used for these experiments. Membrane preparations from these cells were prepared by scraping cells in lysis buffer (25 mM Tris pH 7.4, 5 mM EDTA, 1 μg/mL Aprotinin, 1 μg/mL Leupeptin), followed by centrifugation at 2000 rpm for 5 min. Supernatant was transferred to another centrifuge tube and further centrifuged at 15000 rpm for 25 min. The resulting pellet was resuspended in 200 µL of binding buffer (75 mM Tris, pH 7.4, 2 mM EDTA, 12.5 mM MgCl_2_, 1 μg/mL aprotinin, 1 μg/mL leupeptin) containing 10% glycerol. This membrane preparation was then further homogenized using dounce homogenizer and was then stored at -80 °C until further use.

For radioligand binding, BMDM membranes (5 μg of protein) were incubated with [^125^I]-cyanopindolol ([^125^I]-CYP; 200 pM; PerkinElmer) in binding buffer. Incubations were performed in the presence or absence of propranolol (10 μM) to determine non-specific binding. Reactions were performed in a 250 μL volume and allowed to equilibrate at 37 °C for 1 h before filtering through a glass fiber filter (Whatman GF/C; Brandel). Each filter was washed five times with 5 mL of ice-cold wash buffer (10 mM Tris, pH 7.4, 10 mM EDTA) to remove unbound drug. The amount of total and nonspecific radiolabel bound to membrane was determined on a gamma counter and receptor density was normalized to protein amount of BMDM membranes. All assays were performed in triplicates.

### Cell enrichment

For CD11b^+^ cell isolation from digested hearts, cell pellets were precleared with debris removal solution (130-109-398, Miltenyi Biotec). Briefly, cells were spun at 300 x g for 10 min at 4°C and supernatants were aspirated completely. Cells were resuspended with an appropriate volume of debris removal solution in a 15 mL tube, overlaid by 4 mL of cold 1X PBS and spun as above for phase separation in a swinging bucket centrifuge with full acceleration and full brake. The top two phases containing buffer and interphase were discarded and the tube was filled to a final volume of 15 mL using cold 1X PBS. The solution was mixed gently by inverting three times and spun. Cells were resuspended in freshly prepared isolation buffer (1X PBS, pH 7.2) supplemented with 2 mM EDTA, 0.5% FBS (v/v) and kept on ice until enrichment. After determining cell number, cells were incubated with CD11b microbeads (130-049-601, Miltenyi Biotec), which was diluted in isolation buffer (20 µL per 10 x 10^6^ cells). After 15 min incubation on ice, cells were washed and resuspended in isolation buffer (500 μL/10^8^ cells) for column-based magnetic separation. For neutrophil enrichment, 50 x 10^6^ BM cells were resuspended in chilled isolation buffer (200 µL) containing 20 µL neutrophil biotin antibody cocktail. After 15 min, cells were washed and incubated with anti-biotin microbeads (100 µL) diluted in isolation buffer (400 µL) at 4 °C. After 15 min incubation, cells were washed and resuspended (500 μL/10^8^ cells) in isolation buffer for magnetic column separation (130-097-658, Miltenyi Biotec). Microbead-labelled cells were passed through Miltenyi Biotec MS columns (130-122-727) placed on a MiniMACS™ Starting Kit (130-090-321), and cell fractions were collected as per the cell-type enrichment (CD11b^+^ cells; negative selection and neutrophils; negative selection). Bone marrow-derived monocytes were isolated with the EasySep™ Mouse Monocyte Isolation Kit (19861, STEMCELL Technologies) following the manufacturer's instructions. The purity of the enriched population was subsequently verified by flow cytometric analysis with CD11b BV650 and Ly6C PE-Cy7 antibodies.

### Flow cytometry

Equal volumes of single cell suspension from digested hearts were taken in 5 mL polystyrene round-bottom Falcon tubes and spun at 1000 rpm for 5 min at 4 °C. The cells were incubated in fluorophore labeled antibody (as described in Table S3) diluted in FACS buffer (1X PBS, 10% FBS). After 45 min on ice, cells were washed in FACS buffer. For intracellular staining, BD cytofix/cytoperm fixation/permeabilization solution kit was used as per manufacturer's protocol. Fluorophore-matched IgG antibodies were used as isotype controls. To prevent non-specific antibody binding, FcR blocking was added 15 min prior to primary antibody incubation (Miltenyi Biotec, Germany). The BD LSR-II flow cytometer was used to acquire the cells using FACS diva software, after which the data were analyzed using Flowjo v10.5.0 (BD Biosciences, USA). Absolute cell counts, were attained by normalizing to mg of tissue weight. 1 x 10^6^ cells were used for staining in *ex vivo* experiments and 1 x 10^5^ cells were acquired per sample for in vivo experiments. Fluorophore channel compensation was achieved via UltraComp eBeads™ (Thermo Scientific, USA). Following high FSC/SSC separation, doublets (FSC-A vs FSC-H) were excluded, followed by initial gating for CD45^+^ cells followed by further gating for CD11b^+^ Ly6G^+^ Nu, CD11b^+^ Ly6C^hi/lo^ Mo and CD11b^+^ F4/80^+^ Mac.

### Quantitative reverse transcription polymerase chain reaction (RT-qPCR)

PureLink™ RNA Mini Kit was used to isolate total RNA followed by cDNA synthesis via High-Capacity cDNA Reverse Transcription Kit (Thermo Fisher scientific, USA). Equal amounts of cDNA were used to perform RT-qPCR with PowerUp™ SYBR™ Green Master Mix (Thermo Fisher scientific, USA) with specific primers used in triplicate. Primer3Plus (https://www.primer3plus.com) was used to design the primer sets (Table S1). Quant studio design software (v1.5.1) analysis of RT-qPCR data was performed via comparative CT method (∆∆CT) with glyceraldehyde 3-phosphate dehydrogenase (GAPDH) used for normalization of gene expression.

### *In vitro* assessment of efferocytosis

Nu were isolated from bone marrow as described above and stained for 30 min with 5 µM CellTracker™ Green 5-chloromethylfluorescein diacetate (CMFDA) (Thermo Fisher scientific, USA) in serum-free RPMI-1640 (SFM), after which they were washed in SFM. CMFDA-stained Nu and BMDM were treated with ISO (10 µM, 16 h). BMDM and Nu were co-cultured for 45 min at a 1:3 ratio 41, after which the cells were rinsed with 1X PBS twice to remove unbound Nu. BMDM were subsequently detached using an enzyme-free dissociation buffer (Thermo Fisher scientific, USA), labeled with F4/80 AF647 antibody and processed via flow cytometry as described above.

### Immunofluorescence staining

To detect efferocytosis using immunofluorescence (IF) microscopy, cells were fixed in 4% paraformaldehyde without the use of enzyme-free cell dissociation buffer. Following two washes with 1X PBS, the cells were blocked for 1 h at room temperature in a solution containing 2% BSA (Bovine Serum Albumin) in 1X PBS. Subsequently, they were incubated overnight at 4 °C with rat anti-F4/80 antibody (diluted in 1X PBS + 0.1% BSA). The next day, the cells were washed three times with 1X PBST (1X PBS + 0.05% tween-20) and then incubated with anti-rat AF594 secondary antibody (diluted in 1X PBS) for 1 h. Following another three washes with 1X PBS, the cells were overlaid with 500 μL of 1X PBS and stored in the dark at 4 °C until imaging. Imaging was performed using an EVOS M7000 microscope with GFP and Texas Red filters for CMFDA and AF594, respectively. Percent efferocytosis was quantified using cell counter plugins in the ImageJ software, with each data point per group representative of averaged data from technical duplicates from separate assay wells, with five non-overlapping images/well counted.

### TaqMan-based quantification of micro RNAs

To detect microRNAs (miRNAs), cells were first lysed, and miRNAs were isolated and purified using the miRNeasy Kit (Qiagen) according to the manufacturer's instructions. The purified miRNAs were then quantified using a Nanodrop 2000 spectrophotometer (Thermo Scientific) to determine RNA concentration and ensure sample quality. Subsequently, an equal amount of RNA from each sample was used for complementary DNA (cDNA) synthesis, employing miRNA-specific reverse transcription (RT) primers to generate cDNA tailored for miRNA analysis (Table S5). For quantitative real-time polymerase chain reaction (q-RT-PCR), 200 ng of miRNA-specific cDNA was amplified using stem-loop primers and TaqMan™ Universal Master Mix II (without uracil-N-glycosylase, UNG) on an Applied Biosystems instrument (Table S4). To ensure accurate and consistent miRNA quantification, all expression data were normalized to the expression levels of the U6 small nuclear RNA (snRNA) gene, which served as an internal reference control for the experiment.

### Transfection

The HEK 293T cell line, derived from human embryonic kidney cells, was maintained in complete DMEM as described previously for L929 cells. HEK 293T cells were seeded at a density of 1 × 10^5^ cells per well in 12-well plates to form monolayers overnight. After 12 h of serum starvation, these cells were then transfected with specific microRNA (miRNA) mimics using Lipofectamine RNAiMAX reagent (Thermo Scientific, USA), which was diluted in Opti-MEM medium (Thermo Scientific, USA) following the manufacturer's protocol (Table S5). Twenty-four hours post-transfection, the cells were rinsed with 1X phosphate-buffered saline (PBS) to remove residual transfection reagents and processed for subsequent experimental analyses.

### Prediction of mRNA-miRNA hybrid using IntraRNA program

The IntaRNA software (https://rna.informatik.uni-freiburg.de/IntaRNA/Input.jsp) was engineered for the rapid and precise prediction of RNA-RNA interactions, integrating seed constraints and the accessibility of interaction sites. It is specifically tailored to identify mRNA target sites for non-coding RNAs, such as miRNAs 42, 43. Sequences for Anxa1 mRNA and miR-374b-5p were retrieved from NCBI (https://www.ncbi.nlm.nih.gov/) and miRBase (https://www.mirbase.org/), respectively. RNA-RNA interactions were subsequently predicted using IntaRNA with default parameters.

### Annexin V staining for apoptosis

Enriched neutrophils were analyzed by flow cytometry using specific Annexin V antibody as described previously 44, 45. Briefly, 1 x 10^6^ cells were incubated in Annexin V antibody diluted in 1X binding buffer (10 mM HEPES, 140 mM NaCl, 2.5 mM CaCl_2_, pH 7.4) for 15 min at RT in the dark. Subsequently, 400 µL of 1X Annexin V binding buffer was added to each tube, and samples were immediately acquired using the BD LSR-II instrument followed by analysis using FlowJo software. Approximately 1 x 10^5^ cells were acquired per sample.

### Software and statistical analysis

Graphad Prism 10 (GraphPad Software Inc. USA) was used for statistical analysis. Data were represented Mean ± SEM. Comparison between different groups were carried out by either one-way or two-way ANOVA (repeated measures or mixed model) with Tukey's multiple comparisons test. Unpaired student's t-test was used for comparison between mean of two groups. *p* < 0.05 was considered statistically significant between groups. Biorender.com was used for preparing graphical illustrations.

## Results

### Myeloid cell-specific β2AR deletion improves cardiac function and remodeling post-MI

**Figure 1:**
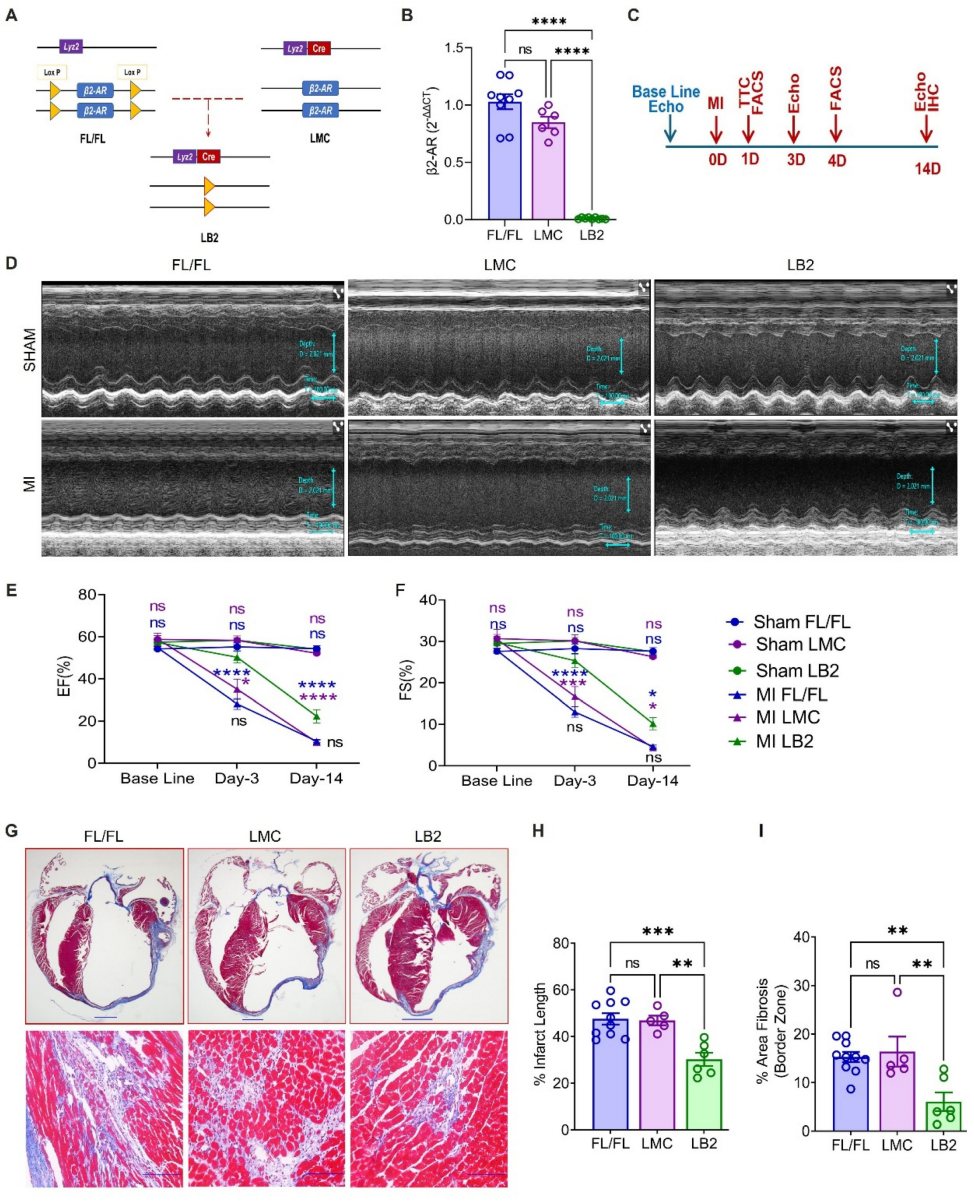
** Myeloid cell-specific β2AR deletion improves cardiac function and remodeling post-MI.** (A) Graphical illustration showing myeloid cell-specific β2AR deletion achieved via crossing β2AR^flox/flox^ (FL/FL) and LysMCre (LMC) mice, resulting in myeloid cell-specific β2AR KO mice (LB2). (B) *β2AR* expression as detected via RT-qPCR in differentiated bone marrow-derived macrophages (n = 9 for FL/FL, n = 6 for LMC, n = 9 for LB2). (C) Study design showing timeline of surgery and echocardiography measurements. (D) Representative echocardiography images (M-mode short axis) of sham (upper panels) or MI (lower panels) FL/FL, LMC or LB2 mice. Histogram depicting percent ejection fraction (%EF, E) and fractional shortening (%FS, F) at baseline, Day-3 and Day-14 post-MI for sham or MI FL/FL (blue), LMC (purple) or LB2 (green) mice (n = 11 for sham FL/FL, n = 9 for sham LMC, n = 12 for sham LB2, n = 10 for MI FL/FL, n = 5 for MI LMC, n = 11 for MI LB2 (day 3), n = 12 for MI FL/FL, n = 9 for MI LMC, n = 6 for MI LB2 (day-14)). (G) Bright field microscopy images of Masson's Trichome (MT) stained LB2, FL/FL and LMC hearts at 14 days post-MI, upper panels (0.8X) scale bar = 1000 µm, lower panels (20X, border zone) scale bar = 100 μm. (H) % infarct length and (I) % area border zone fibrosis at 14 days post-MI (n = 10 for FL/FL, n = 5 for LMC, n = 6 for LB2). Data are Mean ± SEM of independent experiments. ns, non-significant, * *p* < 0.05; ** *p* < 0.01; *** *p* < 0.001, **** *p* < 0.0001, One way ANOVA with Tukey's post-hoc test (B, H, I) or Two-way ANOVA (mixed model) with Tukey's post-hoc test (E, F).

To investigate the role of myeloid cell-specific β2AR in the response to acute cardiac injury, we crossed floxed β2AR (FL/FL) and constitutive myeloid cell-expressing Cre (LysM-Cre) mice to generate myeloid cell-specific β2AR knockout mice (LB2, Figure 1A). Via RT-qPCR, a significant reduction in β2AR expression was observed in total bone marrow (BM) cells from LB2 versus FL/FL and LMC BM cells (Figure S1A), while almost complete ablation of β2AR was observed in LB2 versus FL/FL and LMC bone marrow-derived macrophages (BMDM) (Figure 1B) and β2AR protein levels were significantly reduced in LB2-derived BMDM (Figure S1B). To assess the impact of myeloid cell-specific β2AR deletion on cardiac function and remodeling following injury, we subjected FL/FL, LMC and LB2 mice to sham or MI surgery (Figure 1C). While TTC staining confirmed equivalent acute infarct areas (%) between genotype groups at 1 day post-MI (Figure S1D-E), M-mode echocardiography revealed a significant preservation of several cardiac parameters in LB2 mice versus the control groups at 3 days post-MI (Figure 1D, Figure S1G), including % ejection fraction (EF, Figure 1E) and % fractional shortening (FS, Figure 1F). Both %EF and %FS decreased in LB2 mice at 14 days post-MI versus the 3 day timepoint but were still significantly elevated compared to the control groups without any significant changes in HW/TL ratio among the groups (Figure S1F). Via Masson's trichrome (MTC) staining we observed a significant reduction in % infarct lengths and border zone interstitial fibrosis in LB2 mice as compared to either control line at 14 days post-MI (Figure 1G-I, Figure S1C). Since FL/FL and LMC control mice did not differ in any parameter assessed, FL/FL mice were subsequently used as controls for the remainder of the study to reduce duplicative animal usage. Collectively, these data suggest that the deletion of myeloid cell-specific β2AR enacts early benefits against ischemic cardiac injury to attenuate the acute loss of cardiac function and decrease infarct expansion and fibrosis over time.

### Myeloid cell-specific deletion of β2AR enhances neutrophil efferocytosis

**Figure 2:**
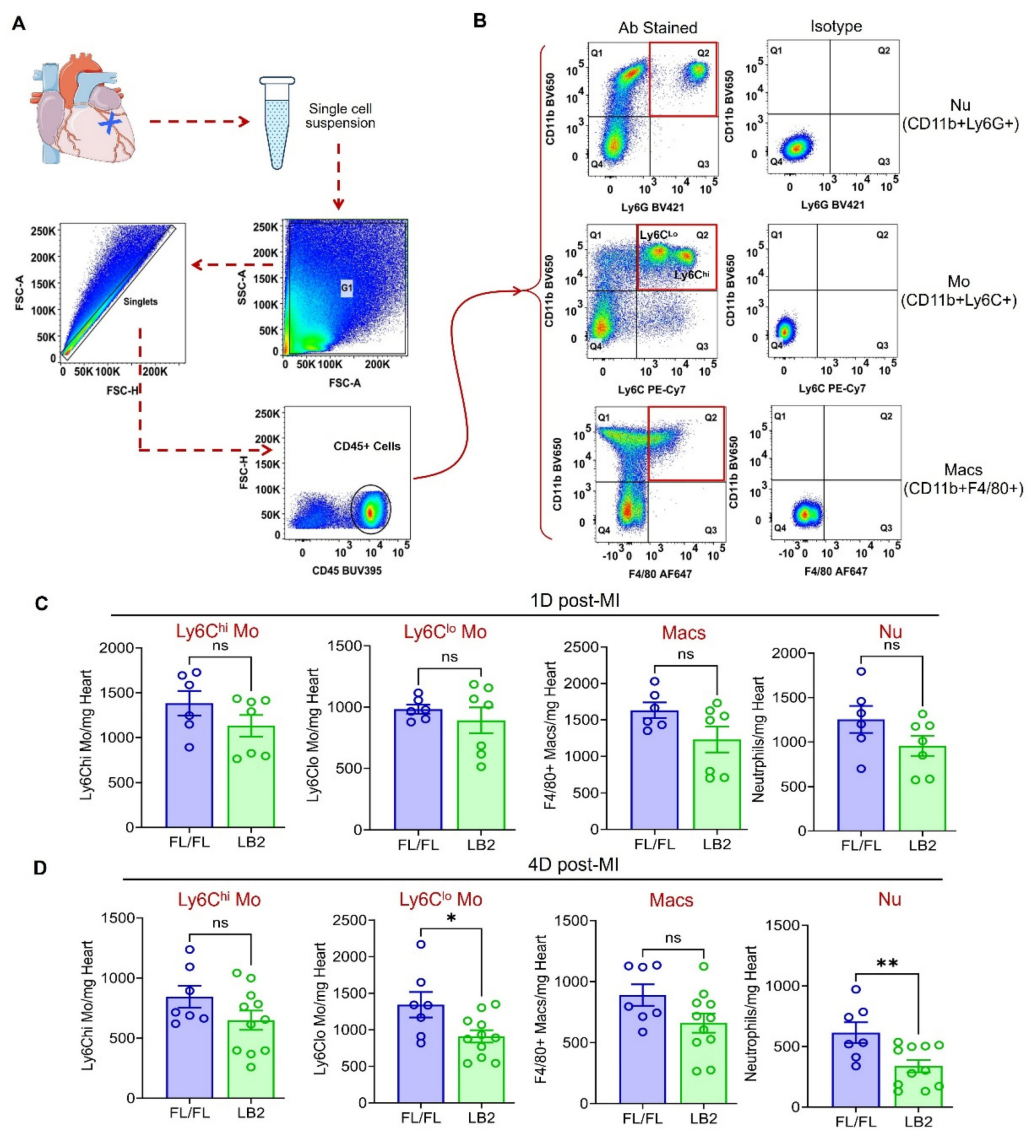
** Myeloid cell-specific deletion of β2AR reduces post-MI cardiac neutrophil clearance.** (A) Flow chart representation of gating strategies used for flow cytometry-based immune cell profiling. (B) Flowjo analysis of representative pseudo-colored dot plots of CD45^+^ gated antibody-stained (left column) Nu (top), Mo (middle) and Mac (bottom) along with corresponding isotype controls (right column). Histograms representing absolute number of immune cell types indicated (CD11b^+^Ly6C^hi^ Mo, CD11b^+^Ly6C^lo^ Mo, CD11b^+^F4/80^+^ Mac and CD11b^+^Ly6G^+^ Nu), normalized per mg of heart weight at 1 day (C) and 4 days (D) post-MI. Data are Mean ± SEM of independent experiments. n = 6 (1 day) or 7 (4 days) for FL/FL, n = 7 (1 day) or 11 (4 days) for LB2. ns, non-significant, * *p* < 0.05; ** *p* < 0.01, Unpaired student's t-test.

With an improved phenotype in mice lacking β2AR specifically in myeloid cells, we next sought to quantify the major myeloid cell populations in the infarcted heart via flow cytometry analysis at 1 and 4 days post-MI. Briefly, all CD45^+^ immune cells were initially gated, then Nu, Mo and Mac were quantified using CD11b^+^Ly6G^+^, CD11b^+^Ly6C^hi/lo^ and CD11b^+^F4/80^+^ gates, respectively (Figure 2A-B). At 1 day post-MI CD11b^+^Ly6Cl^lo^ Mo were unchanged, while CD11b^+^Ly6C^hi^ Mo, CD11b^+^F4/80^+^ Mac and CD11b^+^Ly6G^+^ Nu were each observed to be ~20-25% less in LB2 hearts than FL/FL hearts, which were not statistically significant (Figure 2C). Consistent with these results, CD11b^+^Ly6C^hi^ Mo and CD11b^+^F4/80^+^ Mac also displayed non-significant reductions of ~20-25% in LB2 hearts at 4 days post-MI (Figure 2D). However, at this timepoint a significant reduction in CD11b^+^Ly6G^+^ Nu (~50%), and to a lesser degree CD11b^+^Ly6Cl^lo^ Mo (~30%), were observed in LB2 mouse hearts compared to FL/FL mouse hearts, with no significant differences observed in these myeloid populations in Sham-operated mice (Figure S2A-D) or in the blood post-MI (Figure S2E-H). Altogether, these results indicate that the predominant alteration in β2AR-deficient myeloid cell dynamics in the injured heart is related to enhanced clearance, particularly for Nu, rather than changes in initial accumulation.

**Figure 3:**
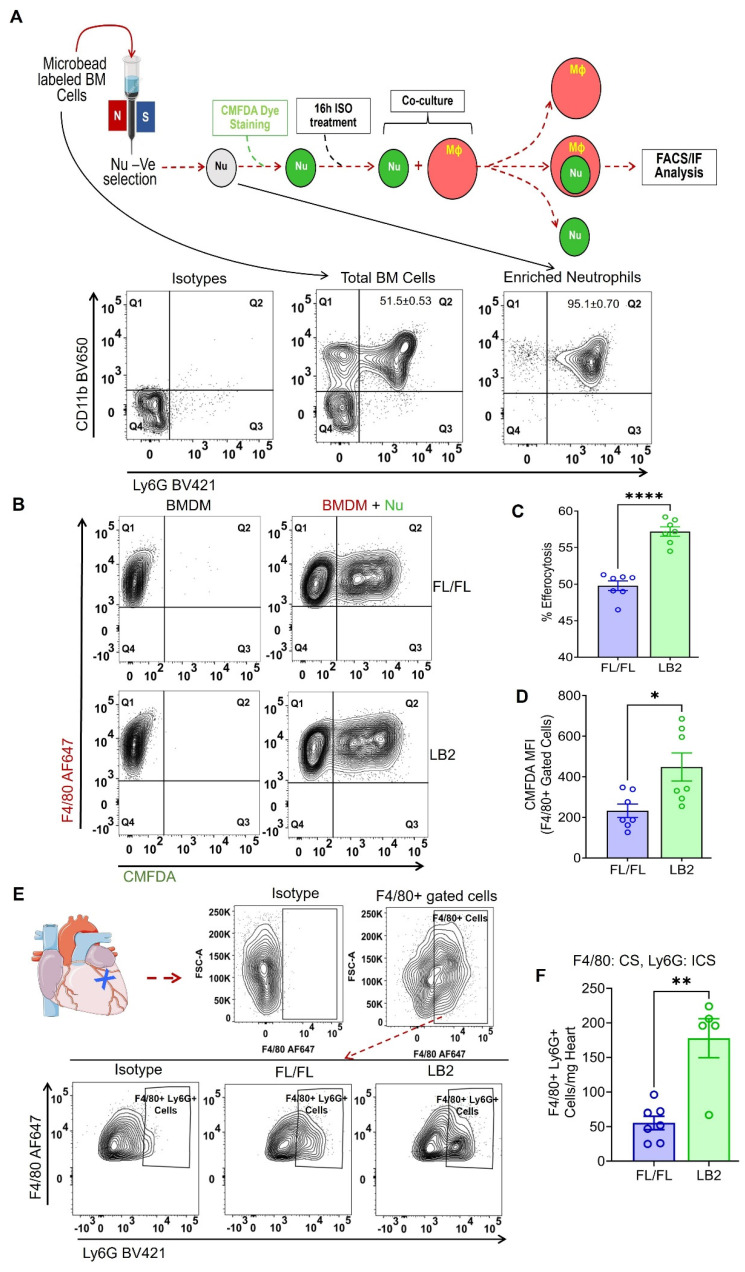
** Myeloid cell-specific deletion of β2AR enhances neutrophil efferocytosis *in vitro*.** (A) Schematic depicting the experimental *in vitro* efferocytosis assay protocol in which Nu were freshly isolated from FL/FL or LB2 BM (as confirmed in FACS panels below), loaded with CMFDA and treated with isoproterenol (ISO, 10 µM) for 16 h prior to incubation with FL/FL or LB2 BMDM (also treated for 16 h with ISO) for 45 min at a 1:3 ratio. (B) FlowJo contour plot of BMDM with (right column) or without (left column) CMFDA^+^ Nu. Quantification of Nu uptake efficiency into BMDM in terms of % efferocytosis (C) and F4/80^+^-gated CMFDA MFI (D), n = 7 each. (E). Flow cytometry analysis of mouse heart digest at 4 days post-MI. Contour plot analysis showing F4/80 AF647^+^ (CS; surface staining) cells (top) and F4/80 AF647^+^ Ly6G BV421^+^ (ICS: intracellular staining) cells in the FL/FL and LB2 mice heart after 4 days post-MI (bottom). (F) Summarized FACS analysis of F4/80^+^ gated Ly6G^+^ (intracellular) cardiac cells at 4 days post-MI from FL/FL (n = 7) or LB2 (n = 5) mouse hearts. Data are Mean ± SEM of independent experiments. ns, non-significant, * *p* < 0.05, ** *p* < 0.01, **** *p* < 0.0001, Unpaired student's t-test used.

To determine whether β2AR deletion increases myeloid cell efferocytosis, we employed an *in vitro* assay in which we freshly isolated bone marrow cells from either LB2 or FL/FL mice to concentrate Nu, the purity of which were assessed by CD11b^+^Ly6G^+^ staining via flow cytometry analysis and consistently found to be ~95% (Figure 3A), or to generate BMDM. To ascertain whether β2AR deletion alters Nu apoptosis rate *in vitro*, we measured Annexin V^+^ staining of the isolated FL/FL and LB2 Nu via flow cytometry at various timepoints up to 72 h post-isolation (Figure S3A-B). No differences in Nu apoptosis were detected at any of the timepoints tested, with ~50% Nu apoptosis achieved by 16 h. To model the post-MI conditions *in vivo* where sympathetic activity is elevated, differentiated BMDM and CMFDA-stained Nu were each treated with 10 µM isoproterenol (ISO) for 16 h, before co-culture within their respective genotype groups at a ratio of 1:3 (BMDM:Nu) for 45 min. Via flow cytometry, the efferocytosis capacity of each of the FL/FL and LB2 myeloid cells was assessed, wherein Nu efferocytosis by BMDM (i.e. F4/80^+^CMFDA^+^ events) was significantly higher in the LB2 versus FL/FL-derived cells (Figure 3B-C). Moreover, the mean fluorescence intensity (MFI) of CMFDA within F4/80^+^-gated LB2 cells was also significantly elevated (Figure 3D) and more co-localization of LB2 CMFDA^+^Nu and F4/80^+^ BMDM were visualized and quantified via immunofluorescence microscopy (Figure S3C-D). To test whether this effect was limited to β2AR-deficient Nu or more generalizable to other types of apoptotic cells, we assessed the efferocytosis of apoptotic human Jurkat T cells by FL/FL and LB2 BMDM, wherein we did not observe a difference (Figure S3E-F), suggesting β2AR deletion is also required in the dying cells to enhance efferocytosis. Importantly, to verify our *in vitro* efferocytosis findings *in vivo*, single cell suspensions were prepared from 4 days post-MI hearts and were further analyzed via FACS (as mentioned in Figure 2A-B) for intracellular Ly6G-staining among F4/80^+^-gated Mac population (Figure 3E), wherein significantly more intracellular Ly6G^+^F4/80^+^ Mac were detected in LB2 versus FL/FL mice (Figure 3F). These data indicate that enhanced Mac-mediated efferocytosis of Nu occurs after cardiac injury in the absence of β2AR in myeloid cells.

### β2AR deletion increases the expression of the efferocytosis protein annexin A1 (AnxA1) in myeloid cells

**Figure 4:**
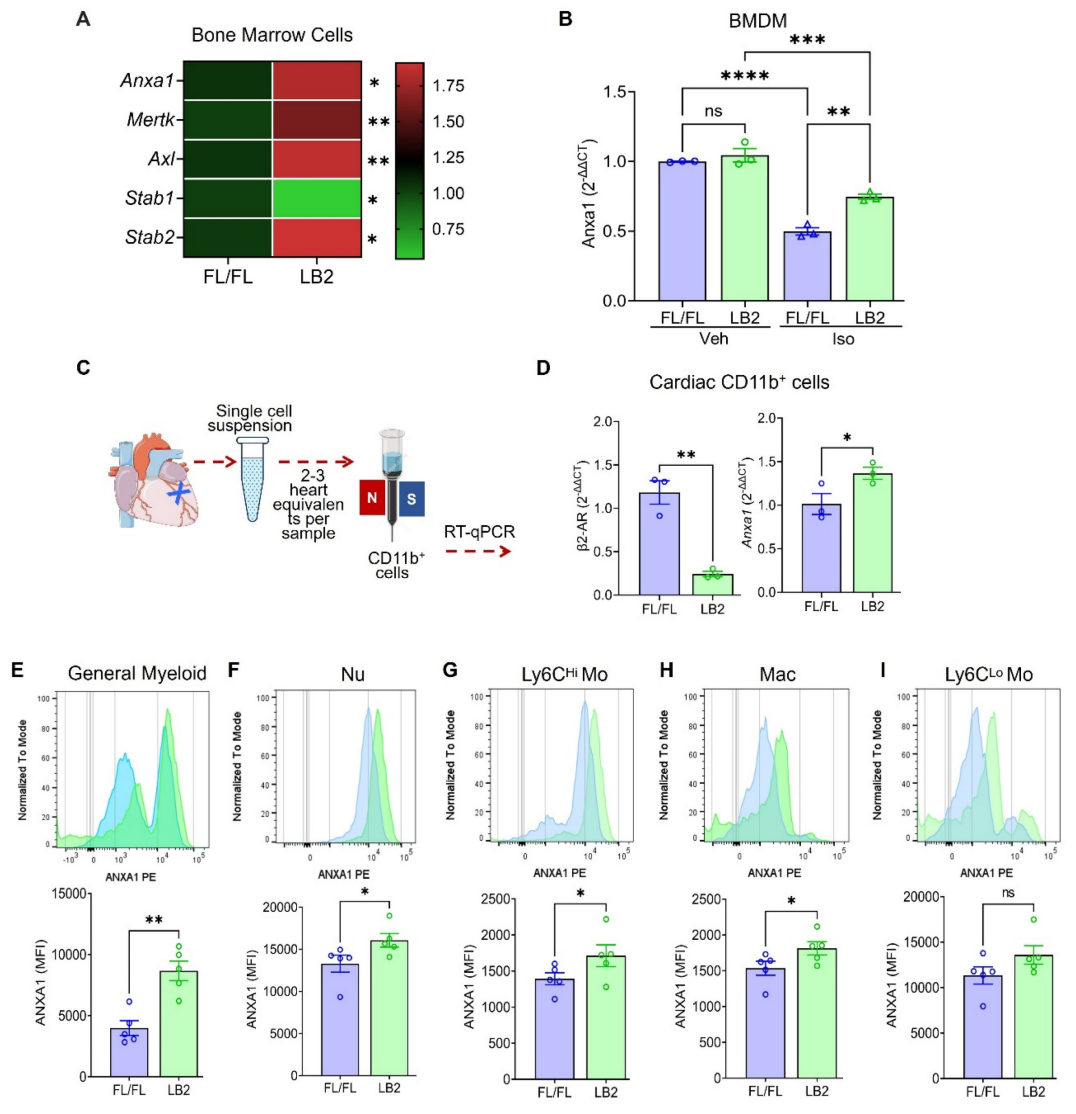
** Annexin A1 expression is increased in β2AR-deficient myeloid cells post-MI.** (A) RT-qPCR screening of various efferocytosis pathway genes was performed on total RNA isolated from whole bone marrow (BM) cells from FL/FL and LB2 mice, with the heatmap representing the differential expression pattern of Anxa1, Mertk, Axl, Stab1 and Stab2 genes between FL/FL and LB2 BM cells. RT-qPCR-detected Anxa1 expression in Veh or ISO-treated (10 µM for 16h) FL/FL versus LB2 BMDM (B, n = 3 each), prepared as described in Figure 3A. (C) Schematic of magnetic microbead column-based cardiac CD11b+ cell enrichment at 1 Day post-MI, after which gene expression of β2AR and Anxa1 were assessed via RT-qPCR (D), n = 3/group. Flow cytometry-based histogram analysis showing comparative AnxA1 protein expression via mean fluorescence intensity (MFI) among various phenotypically distinct myeloid cell types: general CD11b+ myeloid cells (E), Nu (F), Ly6C^hi^ Mo (G), Mac (H) and Ly6C^lo^ Mo (I) at 4 days post-MI FL/FL and LB2 hearts, n = 5/group. Data are Mean ± SEM of independent experiments. ns, non-significant, * *p* < 0.05; ** *p* < 0.01, **** *p* < 0.0001, Unpaired student's t-test.

Since β2AR deletion in myeloid cells increased efferocytosis, we assayed the expression of different efferocytosis-related genes by RT-qPCR in LB2 mouse total bone marrow cells. We observed that *Anxa1*, *Mertk*, *Axl*,* Stab1* and *Stab2*, genes with known roles in efferocytosis, were significantly altered, predominantly increased, in LB2 versus FL/FL bone marrow cells at baseline (Figure 4A). Of these, we confirmed that *Anxa1* expression was also higher in ISO-treated LB2 bone marrow-isolated Nu (Figure S4A-B), prepared as described for our *in vitro* efferocytosis assays (Figure 3). Using BMDM, we observed that, rather than increasing *Anxa1* expression in LB2 cells, ISO treatment actually decreases *Anxa1* expression in FL/FL control cells (Figure 4B), an effect to which LB2 cells are resistant due to the lack of β2AR expression, thereby better preserving *Anxa1* expression under conditions of chronic catecholamine stimulation. To verify these results post-MI, we enriched cardiac CD11b^+^ myeloid cells from LB2 versus FL/FL mouse hearts at 1 day post-MI (Figure 4C), wherein we confirmed loss of *β2ar* expression and significantly higher expression of *Anxa1* (Figure 4D),however there were no changes in *Mertk*, *Axl* or* Stab1* (Figure S4C-E), with *Stab2* undetected in the cardiac CD11b^+^ cells. We next performed flow cytometry analysis at 4 days post-MI to determine which myeloid cell subtypes expressed Anxa1 protein at higher levels in LB2 mice, the timepoint at which we had observed enhanced Nu clearance. Consistent with the RT-qPCR data, MFI analysis indicated significantly more AnxA1 protein expression in LB2 versus FL/FL cardiac CD11b^+^ myeloid cells in general (Figure 4E), and subsequent gating revealed significant increases in Anxa1 expression in CD11b^+^Ly6G^+^ Nu, CD11b^+^Ly6C^hi^ Mo and CD11b^+^F4/80^+^ Mac, but not in CD11b^+^Ly6C^lo^ monocytes (Figure 4F-I).

### β2AR signaling induces miR-374b-5p-mediated repression of *Anxa1*

**Figure 5:**
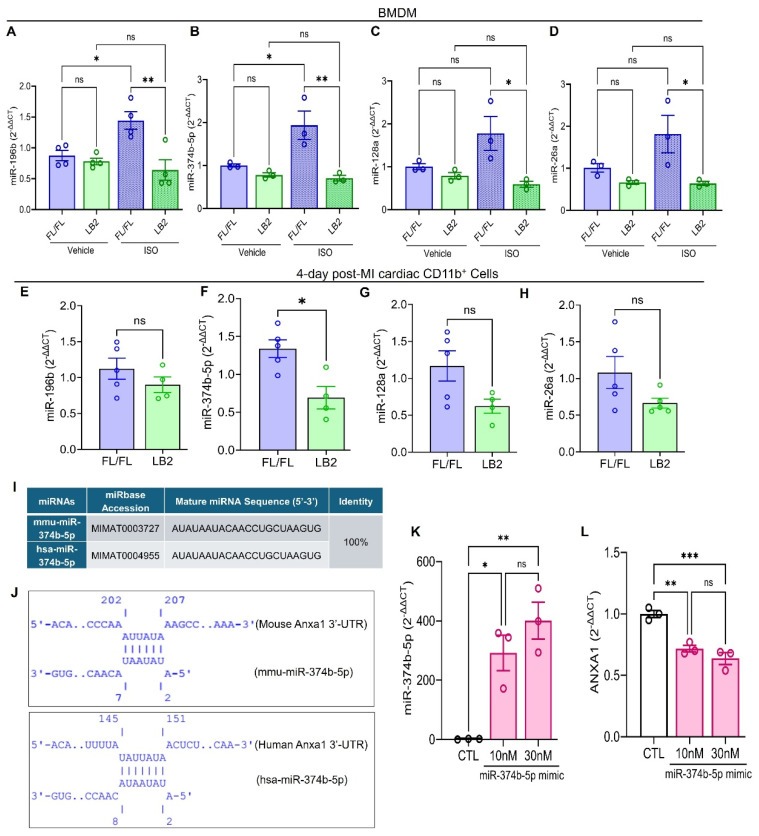
** β2AR signaling induces miR-374b-50-mediated repression of Anxa1.** RT-qPCR screening of miR-196b (A, n = 4 each), miR-374b-5p (B, n = 3 each), miR-128a (C, n = 3 each) and miR-26a (D, n = 3 each) in BMDM treated with vehicle (0.002% L-ascorbic acid) or ISO (10 μM, 16h). RT-qPCR screening of miR-196b (E), miR-374b-5p (F), miR-128a (G) and miR-26a (H) in 4 day post-MI cardiac CD11b^+^ cells, n = 5 FL/FL and n = 4 LB2. For each replicate, a minimum of 5 heart cells were combined. (I) Table showing sequence % identity between mouse (prefixed; mmu-) and human (prefixed; hsa-) miR-374b-5p. (J) Predicated alignment of miR-374b-5p with 3'-UTR of Anxa1. (K) TaqMan^TM^ based RT-qPCR analysis of the effect of miR-374b-5p mimic on miR-374b-5p expression in HEK293T cells. (L) RT-qPCR analysis of the effect of miR-374b-5p mimic on *ANXA1* gene expression in HEK293T cells. Data are Mean ± SEM of independent experiments. ns, non-significant, * *p* < 0.05, ** *p* < 0.01, *** *p* < 0.001, One way ANOVA with Tukey's post-hoc test (A-D, K) or Unpaired student's t-test (E-H).

To gain deeper insight into the mechanism by which *Anxa1* expression is preserved in the absence of β2AR, we focused on micro-RNA (miRNA)-mediated regulation of *Anxa1* due to its inverse relationship with β2AR expression. miRNAs are small, non-coding RNA molecules typically 20-25 nucleotides long that bind messenger RNA (mRNA), usually at the 3'-untranslated region (3'-UTR), leading to its degradation or translational repression 46. With respect to AnxA1, previous studies have reported a handful of miRNAs, specifically miR-128a, miR-196b, miR-26a and miR-374b-5p, capable of regulating its expression by targeting its 3'-UTR 47-50. Consequently, we evaluated the expression of these miRNAs, initially in LB2 versus FL/FL bone marrow-derived macrophages (BMDM) at baseline or in response to stimulation with the βAR agonist ISO. In FL/FL BMDM, ISO treatment increased the expression of each of the miRNAs assayed, with significant elevations observed for miR-196b and miR-374b-5p (Figure 5A-D). In contrast, and consistent with absence of β2AR, none of the miRNAs were upregulated in response to ISO in LB2 BMDM. Notably, miRNA expression was not different in freshly isolated ISO-treated FL/FL versus LB2 bone marrow Mo (Figure S5A-B). However, when we screened cardiac CD11b^+^ cells from LB2 versus FL/FL mice at 4 days post-MI for expression of these miRNAs, we found that miR-374b-5p was significantly lower in LB2 cardiac CD11b^+^ cells (p=0.0105), while miR-128a was moderately, but not significantly, lower in LB2 cardiac CD11b^+^ cells (p=0.0634), and neither miR-196b nor miR-26a were different between LB2 and FL/FL cardiac CD11b^+^ cells (Figure 5E-H). As such, subsequent experiments focused on miR-374b-5p.

Via IntaRNA, we predicted a strong seed sequence of miR-374b-5p between nucleotide 2-7 in mouse *Anxa1* and between nucleotide 2-8 in human *ANXA1* mRNA, with hybridization energies of -2.29 kcal/mol (mouse) and -3.04 kcal/mol (human) (Figure 5I-J). These *in silico* findings suggest that miR-374b-5p specifically regulates AnxA1 mRNA through seed sequences complementarity with its 3'-UTR (Figure S5C). To investigate the regulatory impact of miR-374b-5p on AnxA1, we introduced increasing concentrations of miR-374b-5p mimic in HEK293T cells via transfection, an approach that is inefficient in BMDM. Subsequent evaluation using TaqMan^TM^ RT-qPCR confirmed elevated miR-374b-5p levels (Figure 5K), which corresponded with a significant reduction in *ANXA1* expression (Figure 5L). These data reveal that β2AR stimulation in Mac normally induces miR-374b-5p expression to repress AnxA1 expression, which is relieved upon deletion of β2AR, leading to increased AnxA1 that may contribute to enhanced efferocytosis.

### Anxa1 downregulation reduces efferocytosis and ameliorative cardiac remodeling effects in myeloid cell-specific β2AR knockout mice

**Figure 6:**
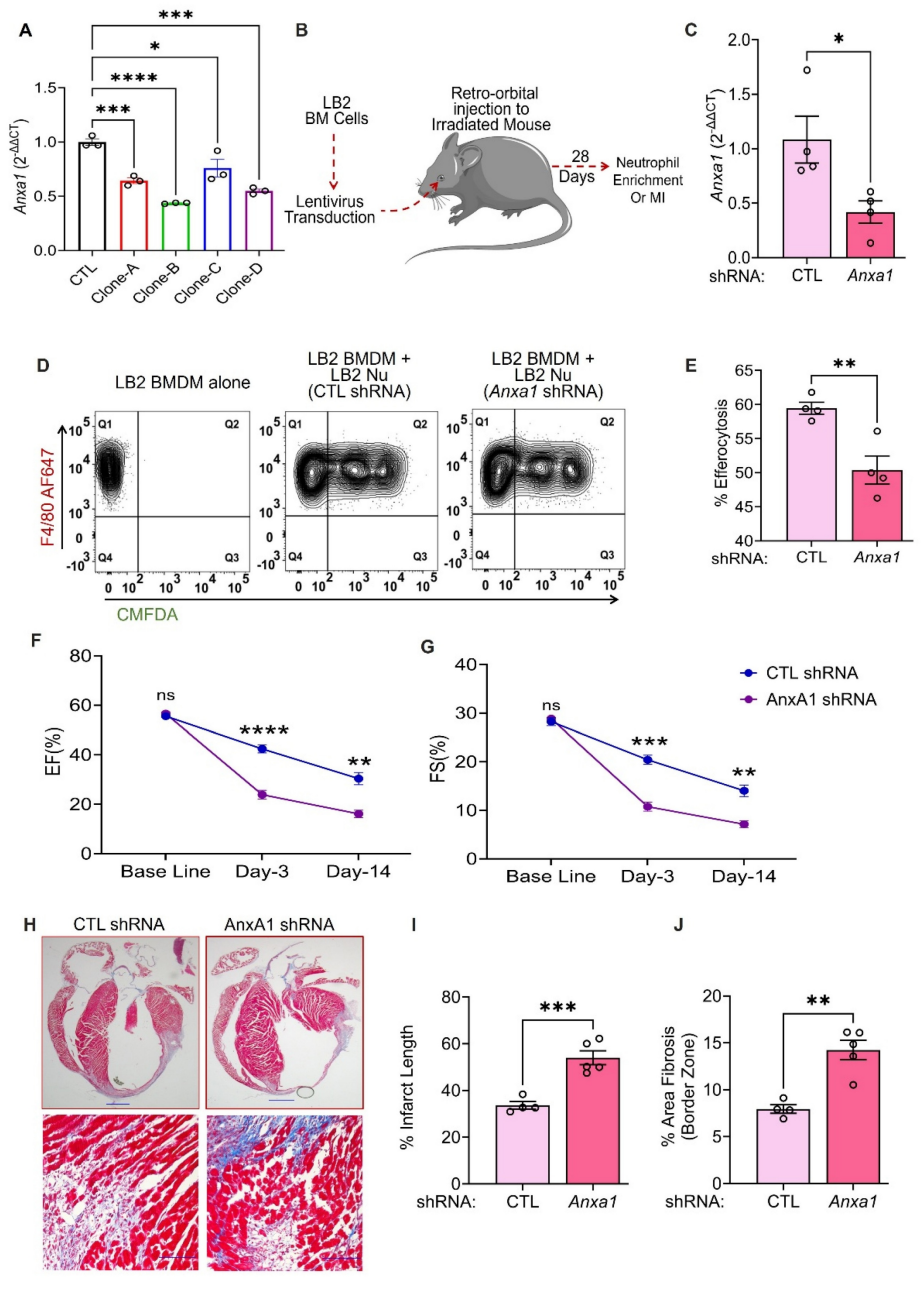
** Anxa1 downregulation reduces efferocytosis and ameliorative cardiac remodeling effects in myeloid cell-specific β2AR knockout mice.** (A) BMDM underwent lentivirus transduction of various clones (A-D) of AnxA1 shRNA versus control (CTL) shRNA for 48 h, after which RT-qPCR was used to assess gene silencing efficiency, n = 3/group. (B) Schematic depicting the experimental design to test the impact of AnxA1 knockdown in LB2 bone marrow-derived Nu. Briefly, LB2 BM cells were transduced with lentivirus containing either CTL shRNA or AnxA1 shRNA (1:1 mixture of clones B and D), after which they were retro-orbitally injected into lethally irradiated LB2 mice to generate bone marrow transplant (BMT) mice featuring LB2 bone marrow with or without AnxA1 knockdown, as confirmed via RT-qPCR analysis after terminal experiments (C), n = 4 (CTL shRNA), n = 4 (AnxA1 shRNA). 4 weeks after BM reconstitution, CTL shRNA and AnaA1 shRNA Nu were enriched from the respective BMT mice using magnetic columns, after which they underwent efferocytosis assays with LB2 BMDM (as described in Figure 3) and flow cytometry analysis, as shown via contour plots (D) of LB2 BMDM incubated without Nu (left) or with CMFDA^+^ Nu from either CTL shRNA (middle) or Anxa1 shRNA (right) LB2 BMT mice. (E) Quantification of CTL shRNA versus Anxa1 shRNA LB2 Nu uptake efficiency into LB2 BMDM in terms of % efferocytosis, n = 4/each group. CTL shRNA and AnxA1 shRNA-transduced LB2 BMT mice were generated as described above and 4 weeks after BM reconstitution underwent MI surgery. Histograms depicting %EF (F) and %FS (G) at baseline, 3 and 14 days post-MI in CTL and AnxA1 shRNA LB2 BMT mice, n = 4 for CTL, n = 6 for AnxA1 shRNA. (H) MT staining of whole heart (top row) displaying infarct size of CTL and AnxA1 shRNA LB2 BMT mice at 14 days post-MI with quantification shown in (I), n = 4 for CTL and n = 5 for AnxA1 shRNA group, scale bar = 1000 µm, and 20X images (bottom row) displaying interstitial fibrosis in the border zone of the post-MI hearts with quantification as % area fibrosis (J), n =4 for CTL and n = 5 for AnxA1 shRNA group, scale bar = 100 µm. Data are Mean ± SEM of independent experiments. ns, non-significant, * *p* < 0.05, ** *p* < 0.01, *** *p* < 0.001, **** *p* < 0.0001, One way ANOVA with Tukey's post-hoc test (A), Unpaired student's t-test (C, E, I, J) or Two-way repeated measures ANOVA with Tukey's post-hoc test (F, G).

Since increased AnxA1 expression is associated with enhanced efferocytosis in myeloid cell-specific β2AR knockout mice, we next tested whether direct downregulation of AnxA1 would restore LB2 myeloid cell efferocytosis efficiency to the level observed in FL/FL cells. Initially, we tested four different lentivirus clones (A-D, Table S2) of mouse *Anxa1*-shRNA in BMDM for their gene silencing efficacy. RT-qPCR analysis showed all the clones were able to significantly decrease *Anxa1* expression in comparison to the scrambled shRNA control (CTL, Figure 6A). Moreover, clones B and D showed maximum efficacy (~50% reduction) and were mixed at a ratio of 1:1 and used in all the subsequent experiments. We have previously used lentivirus-mediated infection of bone marrow cells prior to transplantation as a method to manipulate immune cells responsiveness to cardiac injury *in vivo* 33, thus using this approach we aimed to knockdown *Anxa1* in all cells of hematopoietic origin. Freshly isolated LB2 bone marrow cells were transduced with CTL or AnxA1 (clones B/D) shRNA lentivirus prior to bone marrow transplantation (BMT) into lethally irradiated LB2 mice (Figure 6B). The mice were subsequently used for experiments following at least 4 weeks of BM reconstitution, after which their BM was harvested and *Anxa1* expression confirmed via RT-qPCR (Figure 6C). Nu were enriched from the BM for subsequent efferocytosis assays, as described in Figure 3, by incubating LB2 BMDM with Nu isolated from either LB2 CTL shRNA BMT mice or LB2 *Anxa1* shRNA BMT mice. Efferocytosis of Nu isolated from LB2 BMT mice with *Anxa1* shRNA-mediated knockdown was found to be significantly reduced compared to Nu isolated from LB2 BMT mice with CTL shRNA transduction (Figure 6D-E). These data support the notion that myeloid cell-specific β2AR deletion contributes to enhanced Nu efferocytosis via increased expression of AnxA1.

Since AnxA1 knockdown decreased the *in vitro* efferocytosis of LB2 Nu, we hypothesized that the ameliorative post-MI effects observed in LB2 mice may be prevented via shRNA-mediated knockdown of *Anxa1*. Thus, we generated additional chimeric LB2 mice with CTL versus *Anxa1* shRNA-transduced BM and, 4 weeks following BMT, the mice underwent MI surgery. Cardiac function and remodeling responses were assessed at different timepoints and sustained post-MI *Anxa1* knockdown efficiency confirmed in the BM via RT-qPCR at the terminal timepoint (Figure S6A). Echocardiography showed a significant reduction in both %EF and %FS at 3- and 14 days post-MI in *Anxa1*-shRNA versus CTL-shRNA LB2 BMT mice (Figure 6F-G, Figure S6C), whereas HW/TL remain unchanged (Figure S6B). Moreover, MTC staining revealed that the % infarct length as well as border zone interstitial fibrosis were both significantly increased in the *Anxa1*-shRNA versus CTL-shRNA LB2 BMT mice at 14 days post-MI (Figure 6H-J). Thus, knockdown of *Anxa1* in BM cells was sufficient to prevent the ameliorative effects of myeloid cell-specific β2AR deletion on cardiac function and fibrotic remodeling observed post-MI in LB2 mice.

## Discussion

At steady state, myeloid cells comprise a small portion of the total heart by volume 3, but the release of cytokines and chemokines upon acute ischemic injury leads to a dramatic increase in the recruitment of peripheral myeloid cells, including Nu and Ly6C^hi^ inflammatory Mo 51. Recruitment of these cells to the site of injury occurs quickly, within hours and persisting for several days, allowing the release of proteases and reactive oxygen species to digest and clear the infarct area 51, 52, without which stabilization of the scar cannot occur and rupture ensues. We previously reported that deletion of β2AR in all cells of hematopoietic origin via β2AR KO bone marrow transplantation (BMT) resulted in generalized disruption of immune cell responsiveness to injury, including a decrease in Nu and Mo/Mac accumulation in the heart following MI-induced injury that correlated with impaired wound healing and ultimately cardiac rupture 32, 33. A subsequent study further demonstrated that BMT mice lacking β2AR expression in all cells of hematopoietic origin also displayed impaired recruitment of immune cells, including Nu, Mo and Mac, into the heart in response to chronic catecholamine stimulation, resulting in protection from maladaptive remodeling 53. Together, these studies demonstrated that immune cell-expressed β2AR modulates important facets of acute and chronic cardiac stress, with detrimental versus beneficial outcomes attained in a model-specific context. Further, they suggested that myeloid cell-specific β2AR may be particularly important in these outcomes due to the impact of generalized immune cell β2AR depletion on Nu, Mo and Mac responses to either acute cardiac injury or global catecholamine elevation. However, in contrast to mice with β2AR deletion in all cells of hematopoietic origin 32, in our current study we found that mice with myeloid cell-specific β2AR deletion did not undergo cardiac rupture following MI and in fact displayed improved contractile function compared to the control lines, more so early after injury, but also by 2 weeks post-MI, suggesting better scar stabilization and coinciding with decrease in fibrotic remodeling and infarct length.

Based on our previous reports 32, 33, 53, it was expected that myeloid cell-specific β2AR deletion would result in their reduced recruitment to the heart after acute injury. While we observed a slight reduction in the accumulation of Nu, Mo and Mac within the first 24 h post-MI by flow cytometry, these changes were ultimately not significant. Thus, in contrast to the BMT model of β2AR deletion in all cells of hematopoietic origin, myeloid cell-specific β2AR deletion does not robustly alter their responsiveness or ability to traffic to the ischemic heart immediately following injury. The reasons for this observation are unclear, but may stem from the difference in models, wherein the adoptive transfer of hematopoietic stem cells lacking β2AR expression would result in the clonal expansion of all immune cell lineage precursors.

A previous microarray study that analyzed bulk bone marrow cells lacking both β1AR and β2AR reported thousands of differentially expressed genes involved in myriad ontological functions 54. Thus, global deletion of β2AR in bone marrow stem cell precursors may result in a greater number and/or different alterations in myeloid cell transcriptomes, and ultimately responsiveness to injury, than lineage-specific deletion. Indeed, LysMCre mice were previously shown to induce reporter gene recombination in ~40% splenic Macs, ~40% peripheral Mo and ~80% peripheral Nu versus 100% induction by Vav1-Cre that targets all hematopoietic cells, akin to the BMT model 55, thereby providing a more selective gene deletion strategy with less severe effects that allows myeloid cell recruitment to the heart following injury.

Following cardiac injury, Nu quickly migrate to the infarct area where they become activated and play a dominant role in the acute inflammatory response and early engulfment of cellular debris 56, 57. Although ultimately required to prepare the injury zone for repair, a lack of removal of dead/dying Nu can prolong inflammation and aggravate further tissue destruction, thus efficient clearance of Nu via receptor-ligand mediated Mac efferocytosis 10, 11 is beneficial to transition the post-injury response toward repair and injury resolution 12, 13, 52. While myeloid cell-specific loss of β2AR did not significantly alter Nu apoptosis as assessed *in vitro* or their early post-MI cardiac recruitment *in vivo*, there was a significant reduction in Nu in LB2 hearts at 4 days post-MI, suggesting enhanced clearance. Indeed, expression of several efferocytosis-related genes were found to be increased in the bone marrow cells of mice with myeloid cell-specific β2AR deletion, including *Anxa1*. Additionally, *Anxa1* expression was increased in myeloid cells isolated from the hearts of LB2 versus FL/FL control mice at 1 day post-MI, and AnxA1 protein was increased in cardiac myeloid cells in general, and within Nu, Ly6C^hi^ Mo and Mac specifically, isolated from LB2 hearts at 4 days post-MI.

β2AR-dependent regulation of AnxA1 has not been previously reported, however the inverse relationship in their expression profiles suggested that de-repression of AnxA1 in β2AR-deficient myeloid cells may be due to loss of miRNA-mediated modulation of its expression. Previous work has demonstrated the ability of βAR ligands, including agonists and antagonists, to alter miRNA expression in the heart and various cardiac cells, such as cardiomyocytes and endothelial cells, to regulate the expression of proteins involved in injury-relevant processes like cell survival 58-62. Although βAR-dependent effects on cardiac myeloid cell miRNA have not been studied directly, additional reports showed that AnxA1 expression can be regulated by specific miRNAs including miR-26a and miR-196b in cancer cells 48, 49, miR-128a in leukemic myeloid cells 50 and miR-374b-5p in monocytic THP-1 cells 47. Indeed, our data demonstrated that ISO stimulation of control (FL/FL) BMDM increased expression of these miRs, responses that were completely absent in β2AR-deficient (LB2) BMDM. Despite these changes in BMDM, we did not observe an ISO-mediated change in miR-374b-5p or miR-128a expression in bone marrow sourced monocytes, the reason for which is unknown as they ultimately are differentiated into BMDM, but could suggest that β2AR-dependent regulation of miRNA expression in myeloid cells is influenced by distinct states of maturation or differentiation. Ultimately, in the context of acute cardiac injury, miR-374b-5p was found to be the only one of these miRNAs to be significantly lower in post-MI LB2 cardiac CD11b^+^ cells, coinciding with enhanced *Anxa1 expression*. Indeed, we predicted the binding of miR-374b-5p within the 3'-UTR of both mouse Anxa1 and human ANXA1 mRNA and demonstrated that miR-374b-5p mimic experimentally decreased *ANXA1* expression in HEK 293T cells. These data are corroborated by that of a recent study in which miR-374b-5p mimic was demonstrated to decrease *ANXA1* expression in THP-1 cells and mutation of the 3'-UTR of ANXA1 prevented miR-374b-5p mimic-mediated repression of its luciferase reporter 47. Thus, our findings reveal that β2AR activation in BMDM and CD11b^+^ cardiac myeloid cells increases miR-374b-5p abundance, which in turn represses AnxA1 expression, a mechanism absent in β2AR-deficient myeloid cells that allows their enhanced expression of AnxA1.

The observed increase in AnxA1 expression across multiple myeloid cell types was notable since several studies in non-cardiac fields have demonstrated that myeloid cell (Nu or Mac)-produced AnxA1 acts to enhance Nu apoptosis and subsequent Mac-mediated efferocytosis to resolve inflammation 17-22, 63, consistent with the enhanced Nu clearance observed at 4 days post-MI. With respect to cardiac injury specifically, previous work has shown that AnxA1 is increased in human chronic heart failure patients 64 and that global deletion of AnxA1 in mice worsens inflammation, fibrosis, angiogenesis and cardiac function following ischemic injury 65, 66, whereas treatment of mice with recombinant Anxa1 or its N-terminal-derived peptide Ac-AnxA1_2-26_ promotes cardiomyocyte survival, angiogenesis and better cardiac performance outcomes after ischemia 66-68. These data are consistent with a pro-reparative role for AnxA1 via numerous mechanisms. Here, we found that in the context of acute ischemic cardiac injury, myeloid cell-mediated efferocytosis is also enhanced by AnxA1, with β2AR deletion causing higher myeloid cell-specific AnxA1 expression, increased efferocytosis *in vitro* and enhanced Mac-mediated Nu clearance after cardiac injury *in vivo* and knockdown of AnxA1 expression reversing these effects. However, due to the pleiotropic nature of AnxA1, it is possible that following the pro-efferocytosis effect we observed additional beneficial effects of β2AR deletion-mediated enhancement of myeloid cell-specific AnxA1 expression could directly influence other remodeling processes such as fibrosis, which may warrant follow-up investigation.

While we observed that LysM-Cre-mediated deletion of β2AR resulted in enhanced Anxa1 expression in myeloid cells both before and after cardiac injury, resulting in increased efferocytosis of Nu *in vitro* and post-MI Nu clearance *in vivo*, a limitation of our study stems from the LB2 mouse model itself, in which β2AR deletion in all myeloid cells does not indicate whether its loss in a single myeloid cell subtype is sufficient to drive the phenotype. Lentivirus-mediated downregulation of Anxa1 in LB2 bone marrow prior to transplantation into LB2 mice was able to restore normal efferocytosis *in vitro* and post-MI fibrotic cardiac remodeling and dysfunction *in vivo*, suggesting that the peripherally recruited cells are sufficient for the effect, but still does not provide resolution between Nu and Mo, or whether β2AR deletion in post-MI Mo-derived Mac alone would also be sufficient for the effect. Thus, a logical extension of this work would be the deletion of β2AR using additional models of Cre expression driven by other cell type-selective promoters, such as those more selective for Nu, Mo and/or Mac. Since Anxa1 has been shown to promote ameliorative effects on cardiac remodeling after injury that involve both Nu- and Mac-related processes 66-68, such models would resolve whether the β2AR deletion-mediated effect on Anxa1 expression in a single cell type would be sufficient to improve post-MI injury resolution, or confirm that changes in multiple myeloid cell types are indeed required for maximal benefit.

**Figure 7:**
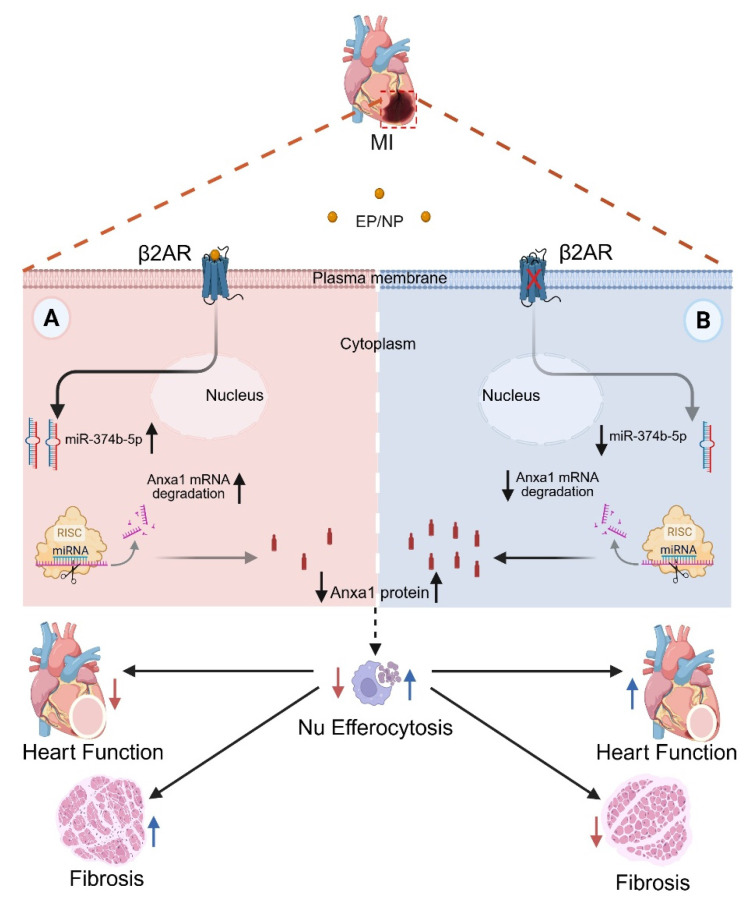
** Mechanism of myeloid cell-specific β2AR deletion-mediated post-MI injury resolution.** Myocardial infarction triggers local ischemic damage and elevated catecholamines (epinephrine/norepinephrine; NE/EP). Stimulation of myeloid cell β2AR increases the abundance of miR-347b-5p leading to degradation of Anxa1 mRNA, resulting in decreased Anxa1 protein expression (A). In the absence of β2AR, miR-347b-5p cannot be induced, leading to de-repression of Anxa1 expression, and ultimately increased Nu efferocytosis (B). This enhanced Nu clearance during the early post-MI period leads to better injury resolution with less fibrosis and infarct expansion and better contractile function.

In conclusion, our data reveal a previously unrecognized role for β2AR in the regulation of myeloid cell-dependent efferocytosis in the heart following injury, wherein its deletion results in loss of miR-374b-5p-mediated repression of Anxa1 expression to allow for enhanced efferocytosis-mediated Nu clearance to limit infarct expansion and improve post-injury cardiac function and fibrotic remodeling (Figure 7). Thus, modulation of β2AR expression or activity specifically in myeloid cells may offer a new approach to limit infarct expansion and preserve healthy myocardium following injury.

## Supplementary Material

Supplementary figures and tables.

## Abbreviations

GPCR: G Protein-Coupled Receptor
β2AR: β2-Adrenergic Receptor
BMT: Bone Marrow Transplant
AnxA1: Annexin a1
Nu: Neutrophils
Mo: Monocytes
Macs: Macrophages
ACS: Acute Coronary Syndrome
MI: Myocardial Infarction
HF: Heart Failure
LCA: Left Coronary Artery
RNA: Ribonucleic Acid
IgGs: Immunoglobulin G
FcR: Fc receptor
GAPDH: Glyceraldehyde 3-Phosphate Dehydrogenase
BMDM: Bone Marrow Derived Macrophages
shRNA: Short hairpin RNA
CTL: Control
miRNA: Micro RNA
cDNA: complementary Deoxyribonucleic Acid
CDS: Coding DNA Sequence
UTR: Untranslated Region
BL: Base Line
ISO: Isoproterenol
RFP: Red Fluorescent Protein
CD: Cluster of Differentiation
M-CSF: Macrophage Colony-Stimulating Factor
FBS: Feta Bovine Serum
DMEM: Dulbecco's Modified Eagle Medium
RPMI-1640: Roswell Park Memorial Institute
EDTA: Ethylenediaminetetraacetic acid
PBS: Phosphate-buffered saline
SFM: Serum Free Medium
ACK buffer: Ammonium-chloride-potassium lysing buffer
RT-qPCR: Real-time polymerase chain reaction
IACUC: Institutional Animal Care and use Committee
MT staining: Masson's Trichrome staining
SEM: Standard Error of the Mean
ANOVA: Analysis of Variance
BMP: Beats Per Minutes
SV: Stroke Volume
EF: Ejection Fraction
FS: Fractional Shortening
CO: Cardiac Output
LV: Left Ventricle
LVAW;s: Left Ventricular Anterior Wall thickness;systole
LVAW;d: Left Ventricular Anterior Wall thickness;diastole
LVPW;s: Left Ventricular Posterior Wall thickness;systole
LVPW;d: Left Ventricular Posterior Wall thickness;diastole
HR: Heart Rate
