# Supplementary material for: Myeloid cell-specific β2-adrenergic receptor deletion improves early cardiac injury resolution via de-repression of Anxa1
Source: Theranostics. 2026 Jan 14;16(8):3959–76. doi: 10.7150/thno.123651 (PMC12905670; doi:10.7150/thno.123651)
Supplement: Supplementary file 1 — Supplementary figures and tables. [file thnov16p3959s1.pdf]

Supplementary Figures

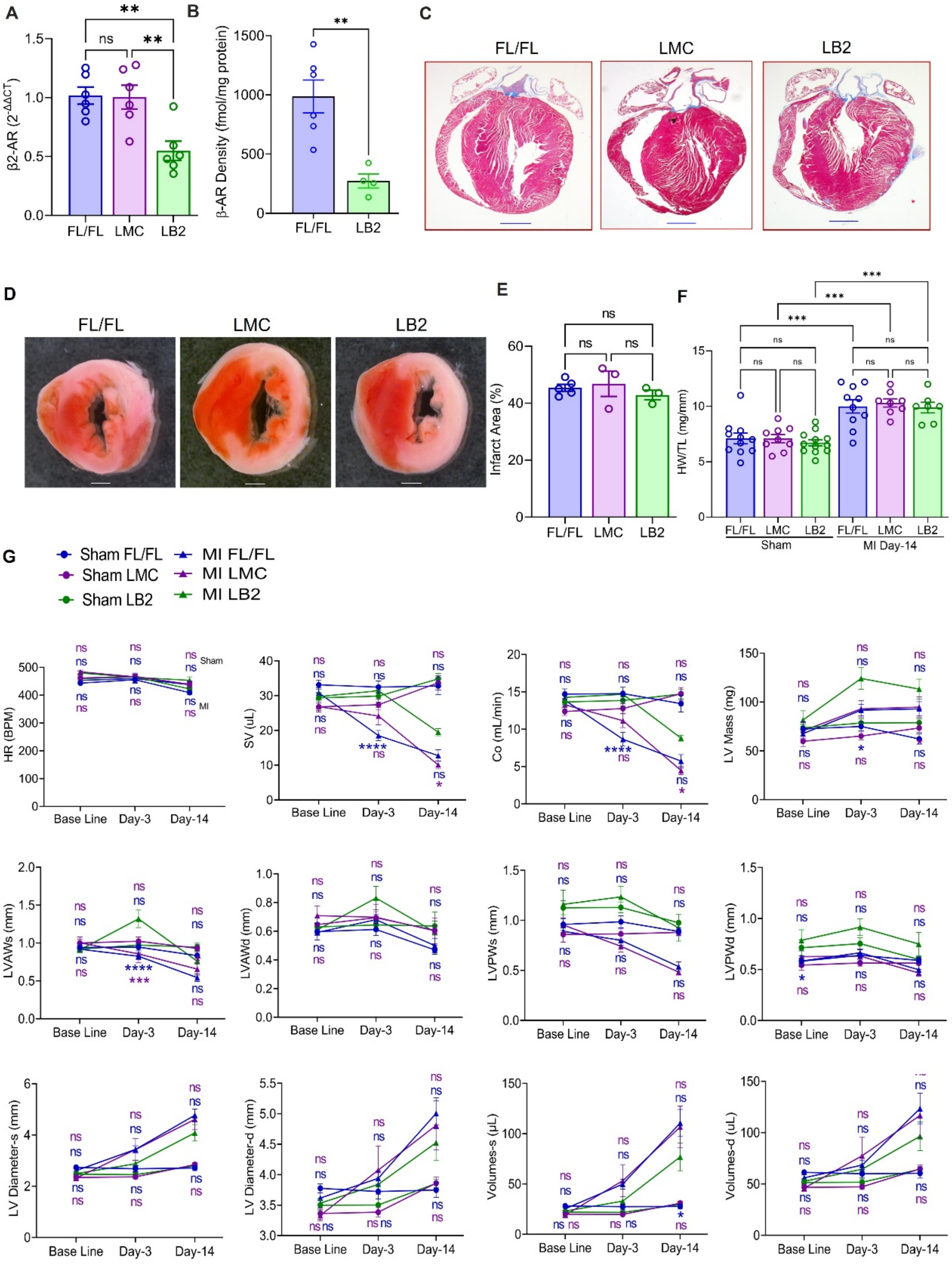

Figure S1. Impact of Myeloid cell-specific β2AR deletion on cardiac function. (A) β2AR expression as detected via RT-qPCR in bone marrow cells (n = 6/group), (B) Graph showing radiolabeled ligand binding to βAR in LB2 versus FL/FL BMDM. (C) Bright field microscopic images (0.8X, scale bar = 1000 µm) of MT staining of sham hearts from LB2, LMC and FL/FL mice. (D) Light microscopic images (0.8X) showing TTC staining of 24 h post-MI hearts of FL/FL, LMC and LB2 mice, scale bar = 1000 µm. (E) % infarct area of heart after 24 h of ischemia (n = 5 for FL/FL, n = 3 for LMC and n = 3 for LB2). (F) Gravimetric quantification analysis of heart weight (HW in mg) normalized to tibia length (TL in mm) at 14-day post-MI. (G) Graphs depicting various echocardiographic parameters (A; HR, B; SV, C; Co, D; LV mass, E; LVAWs, F; LVAWd, G; LVPWs, H; LVPWd, I; LV diameter-s, J; LV diameter-d, K; Volume-s, L; Volume-d) in sham and 3- and 14-day post-MI FL/FL, LMC and LB2 mice. N = 11 for sham FL/FL, n = 9 for sham LMC, n = 12 for LB2, n = 10 for MI FL/FL, n = 5 for MI LMC, n = 11 for MI LB2 (day 3), n = 12 for MI FL/FL, n = 9 for MI LMC, n = 6 for MI LB2 (day 14). Data are Mean ± SEM of independent experiments. ns, non-significant, * $p < 0.05$; ** $p < 0.01$; *** $p < 0.001$, **** $p < 0.0001$. Unpaired student's t-test (B), One way ANOVA with Tukey's post-hoc test (A, E, F) or Two-way ANOVA (mixed model) with Tukey's post-hoc test (G).

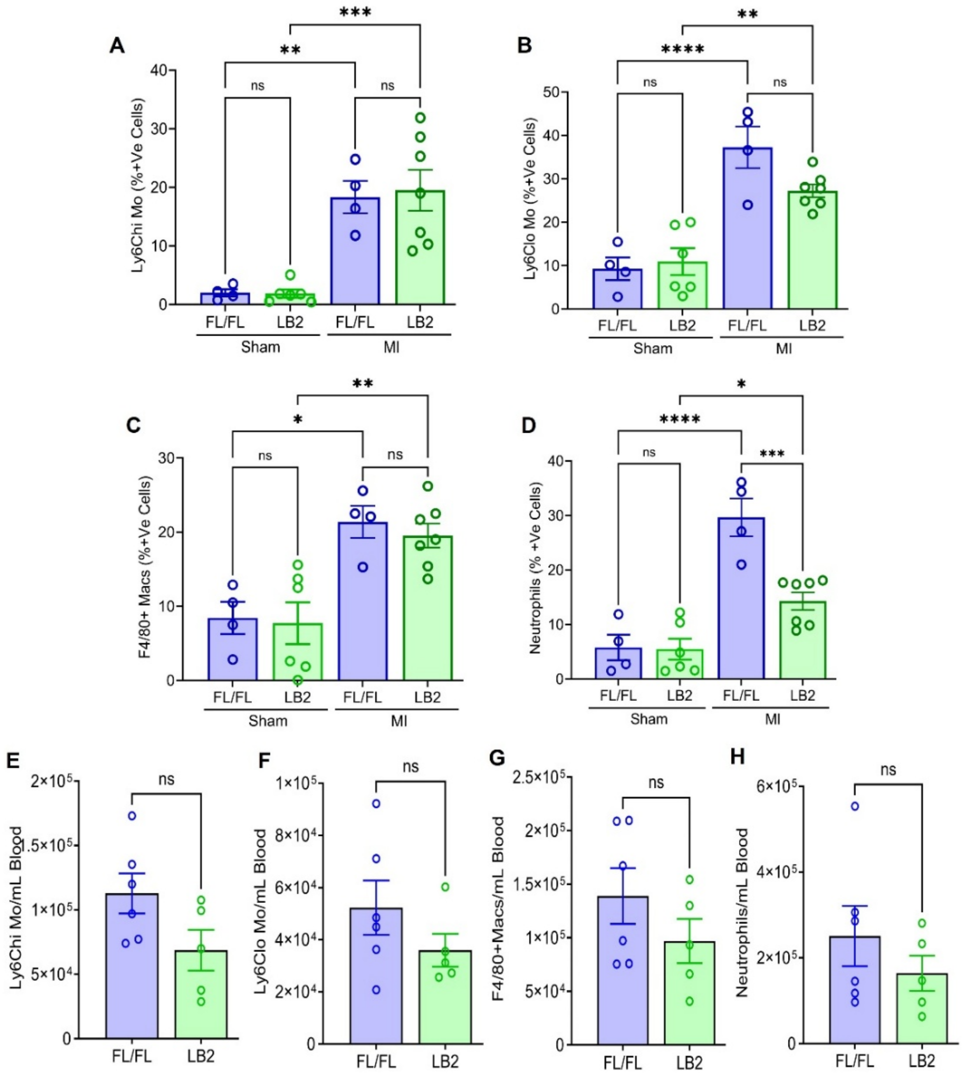

Figure S2. FACS analysis of myeloid cells quantification in sham and MI mice. FACS analysis of 4-day post-Sham or MI heart myeloid cell populations (CD11b^{+} Ly6C^{hi} Mon (A), CD11b^{+} Ly6C^{lo} Mo (B), CD11b^{+} F4/80^{+} Macs (C) and CD11b^{+}Ly6G^{+} Nu (D)) as a percent of CD45^{+} gated cells. Data are Mean ± SEM of independent experiments, Sham FL/FL n = 4, Sham LB2 n = 6, MI FL/FL n = 4, MI LB2 n = 7). ns, non-significant, * $p < 0.05$; ** $p < 0.01$; *** $p < 0.001$; **** $p < 0.0001$. One way ANOVA with Tukey's post-hoc test. FACS analysis of the absolute number of CD11b^{+}Ly6C^{hi} Mon (E), CD11b^{+}Ly6C^{lo} Mon (F), CD11b^{+}F4/80^{+} Mac (G) and CD11b^{+}LyG^{+} Nu (H) in the blood at 4-days

post-MI. Data are Mean ± SEM of independent experiments. N = 6 for FL/FL, n = 5 for LB2. ns, non-significant, Unpaired student's t-test.

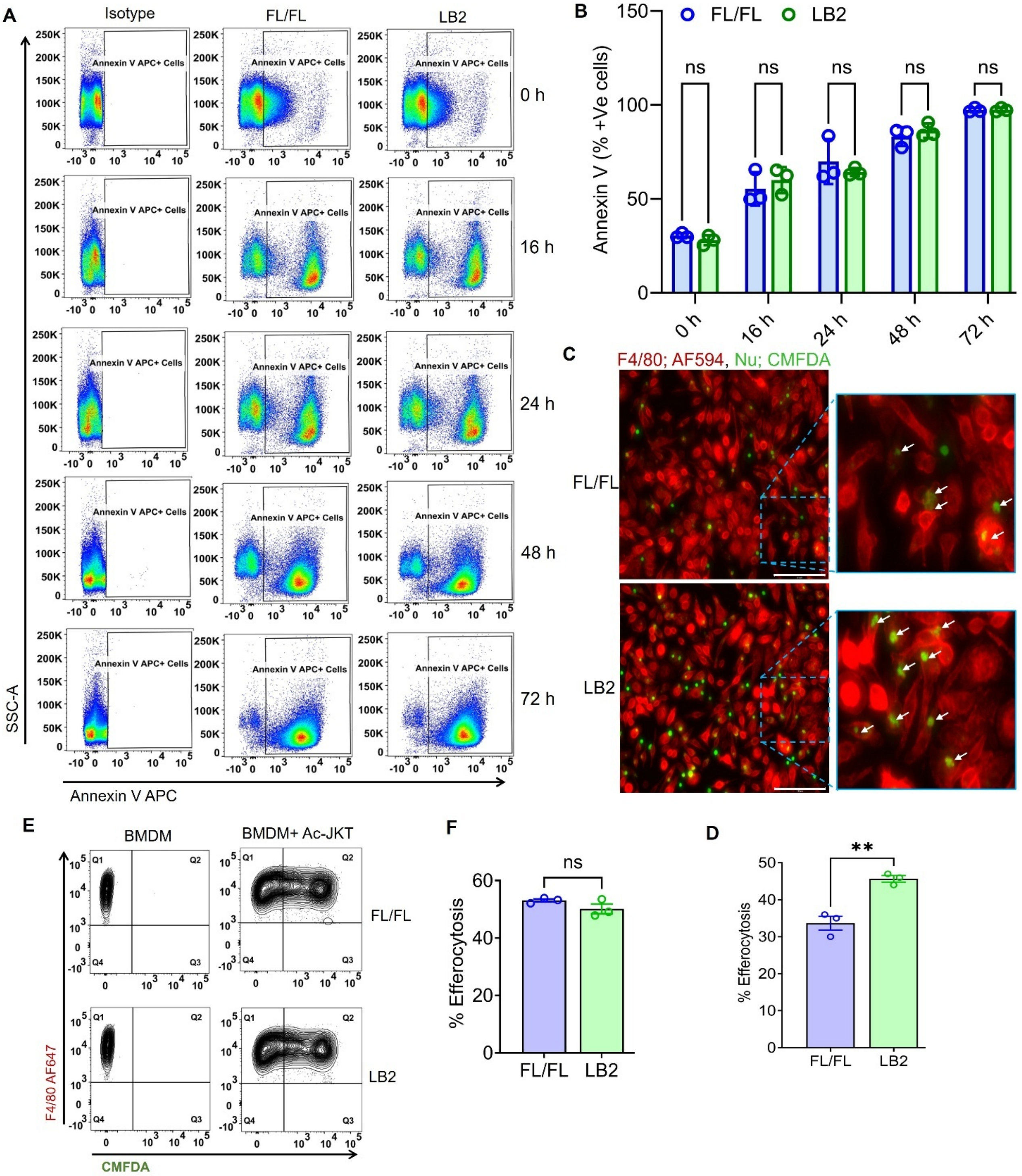

Figure S3. **Myeloid cell-specific deletion of β2AR on neutrophil apoptosis and efferocytosis *in vitro*.** (A) Time course progression of aged-neutrophil apoptosis. Bone marrow neutrophils were isolated and treated with Isoproterenol (ISO) at the concentration of 10 μM followed by stained with

anti-annexin V APC antibody for FACS analysis. Representative pseudocolor dot plot analysis showing rate of apoptosis at 0 h, 16 h, 24 h, 48 h and 72 h. (B) Graph showing Annexin V^+ apoptotic neutrophils (FL/FL and LB2) at various time points, n = 3/group. Data are Mean ± SEM, of three independent experiments. ns, non-significant, 2-way mixed model ANOVA followed by Šidák correction. (C) Immunofluorescence images showing efferocytosis of FL/FL or LB2 Nu (green) by respective BMDM (red) with inset magnification where white arrow showing double positive cells and (D) quantification of corresponding % efferocytosis, scale bar = 125 µm. Data are Mean ± SEM of independent experiments. ns, non-significant, ** $p < 0.01$, Unpaired student's t-test used. (E) Jurkat T cells were stained with CMFDA (Ac-JKT) and were treated with cycloheximide (5 mM) for 24 h to induce apoptosis. Then FL/FL or LB2 BMDM and Ac-JKT were co-cultured and processed further as protocol described under materials section. FlowJo contour plot of BMDM with (right column) or without (left column) CMFDA+ Ac-JKT. (F) Quantification of Ac-JKT uptake efficiency into BMDM in terms of % efferocytosis (n = 3 each. Data are Mean ± SEM of independent experiments. ns, non-significant, Unpaired student's t-test.

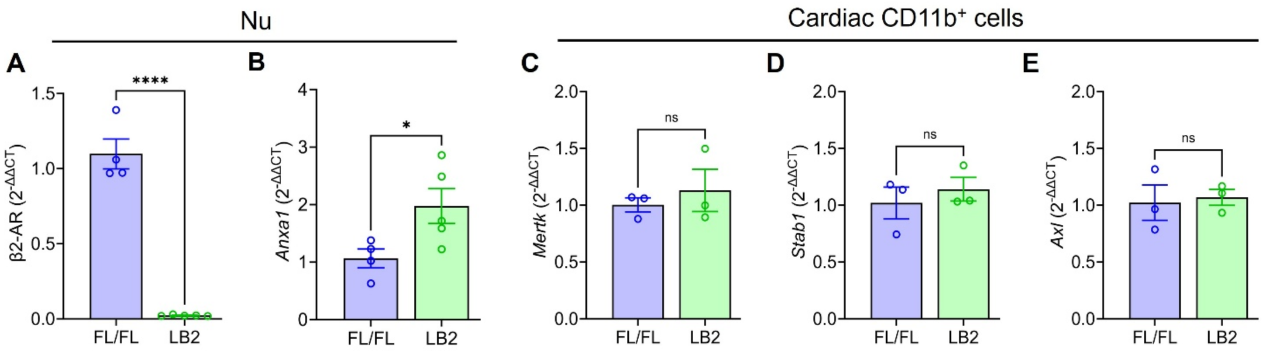

Figure S4. Expression of pro-efferocytosis genes in neutrophils and cardiac $CD11b^+$ cells. RT-qPCR-detected β2AR (A) or Anxa1 (B) expression in ISO-treated FL/FL versus LB2 Nu, n = 4 FL/FL, n = 5 LB2, prepared as described in Fig. 3A. Cardiac $CD11b^+$ cells were isolated at 1-Day post-MI, after which gene expression of *Mertk* (C), *Axl* (D), *Stab1* (E) were assessed via RT-qPCR, n = 3/group. * $p < 0.05$; ns = not significant, Unpaired student's t-test.

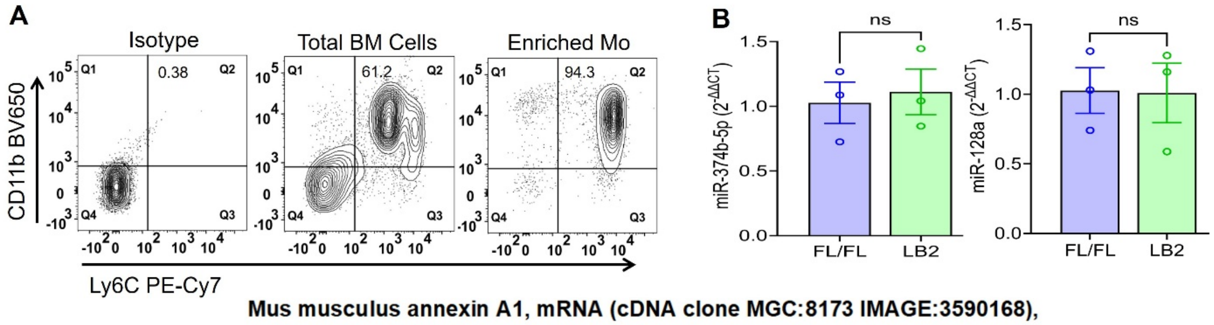

C

Mus musculus annexin A1, mRNA (cDNA clone MGC:8173 IMAGE:3590168), complete cds.

>BC002289.1.

CAAGCAAATACAAAGATACTTCTCTAAAAATGGCAATGGTATCAGAATTCCTCAAGCAAGCCCGTTTTCT
TGAAAATCAAGAACAGGAATATGTTCAAGCTGTAAAATCATACAAAGGTGGTCCTGGGTCAGCAGTGAGC
CCCTACCCTTCCTTCAATGTATCCTCGGATGTTGCTGCCTTGCACAAAGCTATCATGGTTAAAGGTGTGG
ATGAAGCAACCATCATTGACATTCTTACCAAGAGGACCAATGCTCAGCGCCAGCAGATCAAGGCCGCGTA
CTTACAGGAGAATGGAAAGCCCTTGGATGAAGTCTTGAGAAAAGCCCTTACAGGCCACCTGGAGGAGGTT
GTTTTGGCTATGCTAAAAACTCCAGCTCAGTTTGATGCAGATGAACTCCGTGGTGCCATGAAGGGACTTG
GAACAGATGAAGACACTCTCATTGAGATTTTGACAACAAGATCTAACGAACAAATCAGAGAGATTAATAG
AGTCTACAGAGAAGAACTGAAAAGAGATCTGGCCAAAGACATCACTTCAGATACATCTGGAGACTTTCGG
AAAGCCTTGCTTGCTCTTGCCAAGGGTGACCGTTGTCAGGACTTGAGTGTGAATCAAGATTTGGCTGATA
CGGATGCCAGGGCTTTGTATGAAGCTGGAGAAAGGAGAAAGGGGACAGACGTGAACGTGTTCACTACAAT
TCTGACCAGTAGGAGCTTTCCTCATCTTCGCAGAGTGTTTCAGAATTACGGAAAGTACAGTCAACATGAC
ATGAACAAAGCTCTGGATCTGGAACTGAAGGGTGACATTGAGAAGTGCCTCACAACCATCGTGAAGTGTG
CCACCAGCACTCCAGCTTTCTTTGCCGAGAAGCTGTACGAAGCCATGAAGGGTGCCGGAACTCGCCATAA
GGCATTGATCAGGATTATGGTCTCCCGTTCGGAAATTGACATGAATGAAATCAAAGTATTTTACCAGAAG
AAGTATGGAATCTCTCTTTGCCAAGCCATCCTGGATGAAACCAAAGGAGACTATGAAAAAATCCTGGTGG
CTCTGTGTGGTGGAAACTAGACATCCCAACTACTCTGCAAGATTCTGAGGAGAATGTCTCTTAGCTGTTG
TTTTCCTTCTCTTGCATGGGCTTAAGTAGGAAAGTTGCTTTGGCAGATAAGTCTAATTACCTGCTTTGAA
TAATATAGCCTATAAATAGATTTACATCATTACACTGTACAATAGAGAAATACTTGTTTTGTTAATTAT
GTTTATCCCAAATTATAAAGCCCCATAAGCAAGTCACTTTGGTACCATTCCTGAGAAAGAAGTTTACATA
TAATAAAATAAAACAATTTTATAAGACAAAAAAAAAAAAAAAAAAAAAAAAAA

Homo sapiens annexin A1 (ANXA1), mRNA.

>NM_000700.3

AGTGTGAAATCTTCAGAGAAGAATTTCTCTTTAGTTCTTTGCAAGAAGGTAGAGATAAAGACACTTTTTC
AAAAATGGCAATGGTATCAGAATTCCTCAAGCAGGCCTGGTTTATTGAAAATGAAGAGCAGGAATATGTT
CAAACTGTGAAGTCATCCAAAGGTGGTCCCGGATCAGCGGTGAGCCCCTATCCTACCTTCAATCCATCCT
CGGATGTCGCTGCCTTGCATAAGGCCATAATGGTTAAAGGTGTGGATGAAGCAACCATCATTGACATTCT
AACTAAGCGAAACAATGCACAGCGTCAACAGATCAAAGCAGCATATCTCCAGGAAACAGGAAAGCCCCTG
GATGAAACACTGAAGAAAGCCCTTACAGGTCACCTTGAGGAGGTTGTTTTAGCTCTGCTAAAAACTCCAG
CGCAATTTGATGCTGATGAACTTCGTGCTGCCATGAAGGGCCTTGGAACTGATGAAGATACTCTAATTGA
GATTTTGGCATCAAGAACTAACAAAGAAATCAGAGACATTAACAGGGTCTACAGAGAGGAACTGAAGAGA
GATCTGGCCAAAGACATAACCTCAGACACATCTGGAGATTTTCGGAACGCTTTGCTTTCTCTTGCTAAGG
GTGACCGATCTGAGGACTTTGGTGTGAATGAAGACTTGGCTGATTCAGATGCCAGGGCCTTGTATGAAGC
AGGAGAAAGGAGAAAGGGGACAGACGTAAACGTGTTCAATACCATCCTTACCACCAGAAGCTATCCACAA
CTTCGCAGAGTGTTTCAGAAATACACCAAGTACAGTAAGCATGACATGAACAAAGTTCTGGACCTGGAGT
TGAAAGGTGACATTGAGAAATGCCTCACAGCTATCGTGAAGTGCGCCACAAGCAAACCAGCTTTCTTTGC
AGAGAAGCTTCATCAAGCCATGAAAGGTGTTGGAACTCGCCATAAGGCATTGATCAGGATTATGGTTTCC
CGTTCTGAAATTGACATGAATGATATCAAAGCATTCTATCAGAAGATGTATGGTATCTCCCTTTGCCAAG
CCATCCTGGATGAAACCAAAGGAGATTATGAGAAAATCCTGGTGGCTCTTTGTGGAGGAAACTAAACATT
CCCTTGATGGTCTCAAGCTATGATCAGAAGACTTTAATTATATATTTTCATCCTATAAGCTTAAATAGGA
AAGTTTCTTCAACAGGATTACAGTGTAGCTACCTACATGCTGAAAAATATAGCCTTTAAATCATTTTTAT
ATTATAACTCTGTATAATAGAGATAAGTCCATTTTTTAAAAATGTTTTCCCCAAACCATAAAACCCTATA
CAAGTTGTTCTAGTAACAATACATGAGAAAGATGTCTATGTAGCTGAAAATAAAATGACGTCACAAGACA
A

Figure S5. Impact of β2AR signaling on miRNA-374b-5p expression profile in monocytes and its target binding sites on annexin A1 mRNA. (A) Flow cytometry analysis showing purity of freshly enriched bone marrow cell monocytes (Mo). (B) LB2 and FL/FL purified Mo were stimulated with

ISO for 16 h and the expressions of miR-374b-5p and miR-128a were assessed by TaqMan Assay. N = 3/group. Data are Mean ± SEM of independent experiments. ns, non-significant, unpaired Student's t-test. (C) Murine (upper panel) and human (lower panel) annexin a1 gene sequences depicting the 5'-UTR (blue text), coding sequence (CDS, black text), 3'-UTR (red text), start/stop codon (green highlights) and miR-374b-5p target site as predicted by IntaRNA software (yellow highlight).

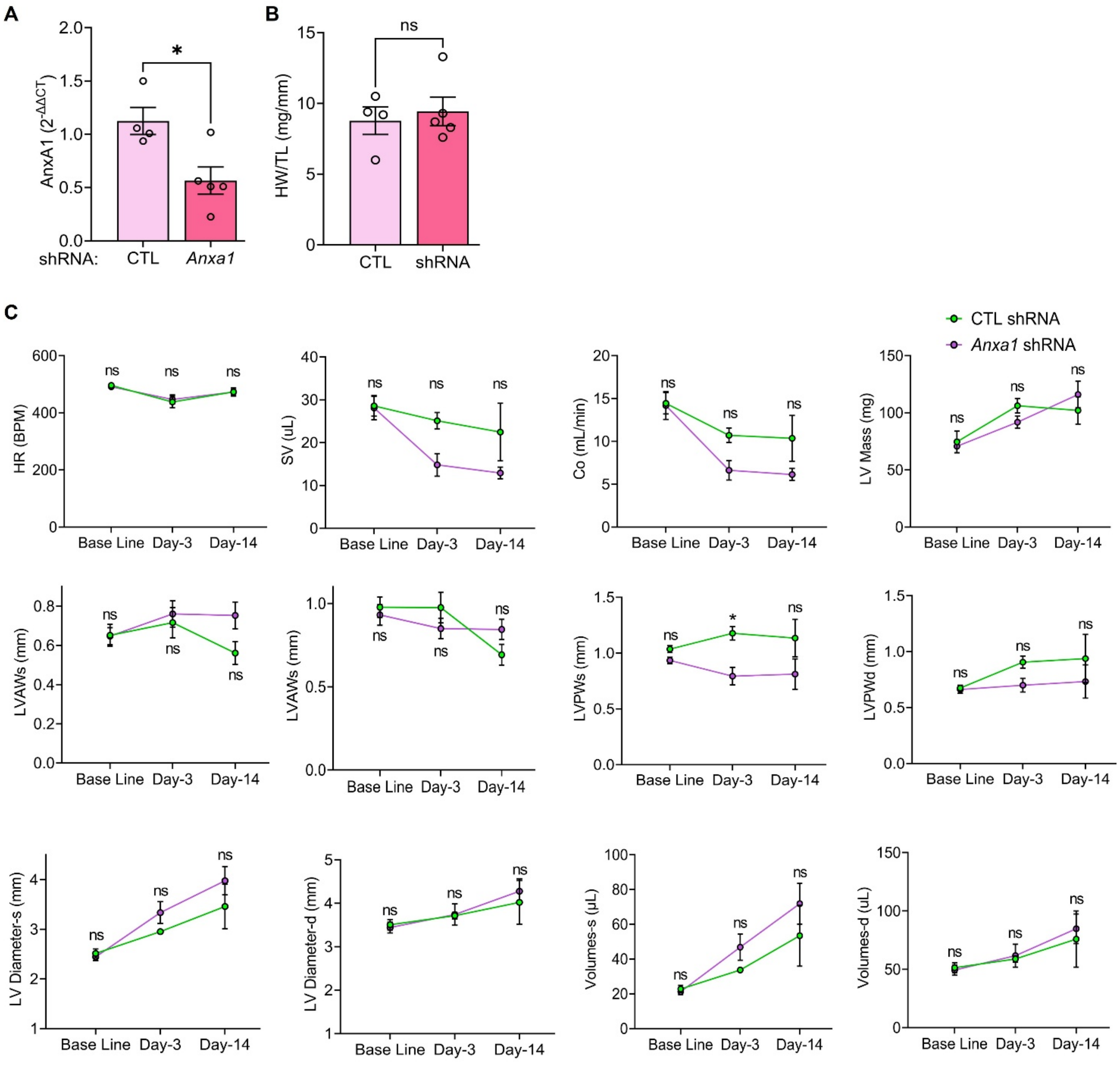

Figure S6. Anxa1 downregulation impaired post-MI cardiac function in LB2 mice. (A) Confirmation of shRNA-mediated *Anxa1* knockdown in LB2 post-MI mice (BM cells) by RT-qPCR, n = 4 for CTL, n = 5 for AnxA1 shRNA. Data were represented as Mean ± SEM of independent experiments, Unpaired student's t-test, * $p < 0.05$. (B) Histogram depicting various parameters (HR, SV, Co, LV mass, LVAWs, LVAWd, LVPWs, LVPWd, LV diameters-s, LV diameters-d, Volumes-s, Volumes-d) of echocardiography measurements at baseline and post-MI at 3- and 14-days in CTL and AnxA1 shRNA LB2 BMT mice. N = 5 for CTL shRNA, n = 6 for AnxA1 shRNA. Data are Mean ± SEM of independent experiments. ns, non-significant, * $p < 0.05$. One way ANOVA with Tukey's post-hoc test.

Supplementary Tables

Table S1: List of primers

Gene Name	Primer Sequence (5’----------3’)	
	Forward	Reverse
Flox	CCAAAGTTGTTGCACGTCAC	GCACACGCCAAGGAGATTAT
Cre	GGCGTTTTCTGAGCATACCT	CTACACCAGAGACGGAAATCCA
Gapdh	AACAGCAACTCCCACTCTTC	CCT GTT GCT GTA GCC GTA TT
Adrb2	AAG AAT AAG GCC CGA GTG GT	GTA GGC CTG GTT CGT GAA GA
Anxa1	TGTCAGGACTTGAGTGTGAA	GCTCCTACTGGTCAGAATTG
Mertk	CAGTCACACCTGAAAAGGAT	AAAAGTTCATCTGGTGATGG
Axl	GAGGAAGAAGGAGACTCGAT	TCCTTCAGCTCTTCACTGAT
Stab1	GACGAGCTCACCTACAAGAC	GGCATTGATACTCAGAAAGG
Stab2	GATGAGCCATACACCATTTT	TCTGACGAGTTCCAGAAGTT
hANXA1	TAAGGGTGACCGATCTGAGG	ACGTCTGTCCCCTTTCTCCT
hTPT1	GGGCTGCAGAACAAATCAAG	CATCCTCACGGTAGTCCAATAG

Table S2: Details of Mouse AnxA1 shRNA RFP-Lentivirus plasmids target sequences with TR30030 (Origene,) backbone with 5’TCAAGAG3’ as loop

AnxA1 shRNA Clone	Sequence
HC108601A	GGCAATGGTATCAGAATTCCTCAAGCAGG
HC108601B	GGCCAAAGACATCACTTCAGATACATCTG
HC108601C	TGACCGTTGTCAGGACTTGAGTGTGAATC
HC108601D	AAGCCATCCTGGATGAAACCAAAGGAGAC

Table S3: List of antibodies used for flow cytometry and microscopy

Antibodies	Catalog	Fluorophore	Manufacturer	Dilution/Concentration
CD45	564279	BUV395	BD, NJ, USA	1:100
CD11b	563402	BV650	BD, NJ, USA	1:50
Ly6C	128018	PE-Cy7	BioLegend, CA, USA	1:300
Ly6G	562737	BV421	BD, NJ, USA	1:100
F4/80	MF48021	Alexa Fluor 647	Invitrogen, CA, USA	1:100
AnxA1	ab225512	PE	abcam, Cambridge, UK	1:50
Rat IgG2b	563560	BUV395 k	BD, NJ, USA	1:100
Rat IgG2a	563236	BV650 k	BD, NJ, USA	1:100
Rat IgG2c	400721	PE-Cy7 k	BioLegend, CA, USA	1:300
Rat IgG2a	562602	BV421	BD, NJ, USA	1:100
Rat IgG2a	R2A21	Alexa Fluor 647 k	Invitrogen, CA, USA	1:50
Rab mAb	ab209478	PE	abcam, Cambridge, UK	1:50
F4/80 Monoclonal Antibody	14-4801-82	N/A	Invitrogen, CA, USA	5ug/mL
Donkey anti-Rat IgG	A-21209	Alexa Fluor 594	Thermo Scientific, MA, USA	1:500
Annexin V	20-6409	APC	Tonbo Biosciences, CA, USA	1:20

Table S4: List of reagents, chemicals, and kits

Name	Catalog	Manufacturer
RPMI-1460	10-040-CV	Corning, NY, USA
DMEM	10-117-CV	Corning, NY, USA
FBS	900-108	GeminiBio, CA, USA
PSF	400-101	GeminiBio, CA, USA
PBS	PB399-20	Thermo Fisher Scientific, MA, USA
L-Ascorbic acid	A5960	Sigma Aldrich, MO, USA
Isoproterenol	16504	Sigma Aldrich, MO, USA
Collagenase	L5004159001	Worthington, NJ, USA
DNase-I	10104159001	Sigma Aldrich, MO, USA
Hyaluronidase	H3506	Sigma Aldrich, MO, USA
EDTA	351-027-101	Quality Biologicals, MD, USA
ACK lysing buffer	118-156-721	Quality Biologicals, MD, USA
Live/Dead Aqua	L34966	Thermo Fisher Scientific, MA, USA
PureLink™ RNA Mini Kit	12183018A	Thermo Fisher Scientific, MA, USA
High-Capacity cDNA Reverse Transcription Kit	4368813	Thermo Fisher Scientific, MA, USA
PowerUp™ SYBR™ Green Master Mix for qPCR	A25741	Thermo Fisher Scientific, MA, USA
CD11b Microbeads	130-097-142	Miltenyi Biotec, Bergisch Gladbach, Germany
Neutrophil Isolation Kit, mouse	130-097-658	Miltenyi Biotec, Bergisch Gladbach, Germany
Opti-MEM™ I Reduced Serum Medium	31985070	Thermo Fisher Scientific, MA, USA
Lipofectamine™ RNAiMAX Transfection Reagent	13778150	Thermo Fisher Scientific, MA, USA
miRNeasy Kit	217004	Qiagen, Germany
TaqMan™ Universal Master Mix II, no UNG	44-400-40	Thermo Fisher Scientific, MA, USA
EasySep™ Mouse Monocyte Isolation Kit	19861	STEMCELL Technologies, BC, Canada

Table S5: TaqMan Assay primers, miRNA mimics and inhibitors

Name	Catalog	Assay ID	Manufacturer
U6 snRNA	-	001973	Thermo Fisher Scientific, MA, USA
mmu-miR-374-5p	4427975	001319	Thermo Fisher Scientific, MA, USA
hsa-miR-196b	4427975	002215	Thermo Fisher Scientific, MA, USA
hsa-miR-128a	4427975	002216	Thermo Fisher Scientific, MA, USA
mmu-miR-26a-2	4440886	463227_mat	Thermo Fisher Scientific, MA, USA
mirVana™ miRNA Mimic, Negative Control#1	4464061	-	Thermo Fisher Scientific, MA, USA
mmu-miR-374-5p mimic	4464066	MC11339	
